# Heterointerface Engineering of Bismuth Nanosheets/Nitrogen‐Doped Carbon Nanoleaves Enables High‑Performance Electrochemical Dechlorination

**DOI:** 10.1002/advs.75448

**Published:** 2026-04-29

**Authors:** Bohan Liu, Feifei Pang, Xingtao Xu, Duo Yang, Xi Yu, Sikan Yang, Jiarui Hao, Nithima Khaorapapong, Zhichang Xiao, Xiaoxian Zhao, Yusuke Yamauchi, Shuaihua Zhang

**Affiliations:** ^1^ Department of Chemistry College of Science Hebei Agricultural University Baoding Hebei China; ^2^ College of Science and Technology Hebei Agricultural University Cangzhou Hebei China; ^3^ Marine Science and Technology College Zhejiang Ocean University Zhoushan Zhejiang China; ^4^ Department of Materials Process Engineering Graduate School of Engineering Nagoya University Nagoya Japan; ^5^ Department of Chemistry School of Science Tianjin University Tianjin China; ^6^ Department of Chemistry Faculty of Science Khon Kaen University Khon Kaen Thailand; ^7^ Department of Plant & Environmental New Resources and Graduate School of Green‐Bio Science Kyung Hee University Yongin‐si Gyeonggi‐do South Korea; ^8^ School of Chemical Engineering and Australian Institute for Bioengineering and Nanotechnology (AIBN) The University of Queensland Brisbane Queensland Australia

**Keywords:** bismuth nanosheets/nitrogen‐doped carbon nanoleaves, capacitive deionization, chloride ion capture, heterointerface engineering, reversible phase transformation

## Abstract

Chloride ion capture from industrial wastewaters presents a persistent challenge, constrained by the corrosive characteristics of accumulated Cl^−^ and the limitations of conventional dechlorination technologies. Herein, we introduce a MOF‑mediated 2D‑on‑2D heterointerface engineering strategy to fabricate bismuth nanosheets coupled with nitrogen‐doped carbon nanoleaves (BiNS/NCL). As a Faradaic dechlorination anode in capacitive deionization (CDI), the rationally designed BiNS/NCL heterointerface delivers an impressive chloride adsorption capacity of 108.7 mg g^−1^ with an adsorption rate of 24.8 mg g^−1^ min^−1^, along with remarkable charge efficiency (86.9%) and low energy consumption (0.39 Wh g^−1^). The BiNS/NCL electrode also demonstrates exceptional chloride selectivity over competing anions and maintains stable performance over extended cycling. Integrated ex situ/in situ characterizations and density functional theory simulations reveal that a built‑in electric field formed at the BiNS/NCL interface thermodynamically favors chloride electrosorption, accelerates ion transport kinetics, and stabilizes the reversible Bi/BiOCl phase transformation. This study elucidates an interface‐mediated dechlorination mechanism and provides a generalizable heterointerface‑engineering strategy for energy‑efficient electrochemical dechlorination systems.

## Graphical abstract

Bismuth nanosheets integrated with nitrogen‐doped carbon nanoleaves are constructed via a 2D‐on‐2D heterointerface engineering strategy. The resulting BiNS/NCL electrode delivers high chloride adsorption capacity, fast kinetics, and stable cyclability. A built‐in electric field at the heterointerface promotes chloride electrosorption and stabilizes the reversible Bi/BiOCl phase transformation, offering a generalizable platform for energy‐efficient dechlorination.

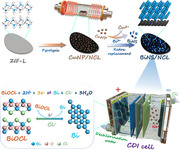

## 1 Introduction

The transition toward sustainable manufacturing in water‑intensive and high‑precision industries, such as microelectronics, precision engineering, and clean energy systems, is critically constrained by the challenges associated with industrial wastewater management, in which the accumulation of chloride ions (Cl^−^) presents a particularly persistent and economically consequential challenge [1, 2, 3]. Notably, Cl^−^ acts as an aggressive corrosion agent, capable of penetrating protective oxide films and deactivating corrosion inhibitors, thus accelerating both corrosion and scaling processes [4, 5, 6]. Conventional dechlorination technologies, such as physical precipitation [7], membrane separations [8], and ion‑exchange processes [9, 10], often involve high energy consumption, complex operational requirements, or the generation of secondary waste, all of which undermine their economic feasibility and environmental sustainability [11, 12]. Capacitive deionization (CDI) has emerged as a promising electrochemical alternative for the sustainable capture and storage of chloride ions, characterized by low energy consumption, high separation efficiency, and environmental compatibility [11, 12, 13]. Unlike traditional methods, CDI leverages an electrosorption‑dominated mechanism that circumvents the generation of secondary chemical pollutants and enables facile electrode regeneration through polarity reversal [14, 15]. These advantages, together with its intrinsic scalability and cycling stability, position CDI as a sustainable pathway for selective chloride management in industrial wastewater treatment.

Electrodes function as the critical operational components in CDI systems, governing both the desalination capacity, separation selectivity, and cycling stability, all of which are particularly important for efficient Cl^−^ capture. To date, a limited number of materials have demonstrated effective Cl^−^ capture capabilities, among which Ag/AgCl [16, 17], layered double hydroxides (LDHs) [18, 19], covalent organic frameworks (COFs) [20, 21], and Bi/BiOCl [22, 23] represent the most widely investigated candidates. However, Ag/AgCl, despite its favorable electrochemical reversibility, suffers from high costs and significant volumetric changes during repeated cycling [24, 25]. LDHs, while offering favorable structural tunability, face limitations related to moderate electrical conductivity and long‐term cycling stability [26, 27]. COFs, recognized for their well‐defined porous architectures and chemically tailorable active sites, generally demand precise and stringent synthesis protocols while also exhibiting intrinsically low electrical conductivity [28, 29]. In this context, bismuth‑based materials (Bi/BiOCl) have attracted considerable interest for Cl^−^ capture applications owing to their inherent Cl^−^ affinity and reversible redox‑driven phase transformation [30, 31]. Nevertheless, their implementation in practical CDI systems remains fundamentally constrained by a kinetic‑capacity‑stability trilemma: sluggish ion diffusion limits dechlorination rate, insufficient accessible active area restricts dechlorination capacity, and repetitive volumetric changes during Bi ↔ BiOCl cycling compromise long‑term structural stability [22, 23]. To address these interconnected challenges, integrating heterointerface engineering with conductive nanostructured supports has emerged as a promising materials‑design strategy [22, 23]. The established Bi/conductive nanostructured heterointerface, driven by the work‐function mismatch between Bi and the conductive supports, generates built‐in electric fields that lower the energy barrier for chloride adsorption and accelerate interfacial charge transfer. Simultaneously, the conductive nanostructured supports spatially confine Bi/BiOCl active phases, shorten ion‑transport pathways, and buffer mechanical strain during cycling.

Metal‑organic frameworks (MOFs), with their structural and compositional tunability, serve as ideal precursors for creating such conductive nanostructured supports [32, 33]. Upon controlled pyrolysis, MOFs transform into heteroatom‑doped carbon architectures with high conductivity, hierarchical porosity, and tailored surface chemistry, which is critical for stabilizing dispersed bismuth active sites and regulating interfacial charge distribution [23]. Motivated by these considerations, we developed a 2D‑on‑2D heterointerface engineering strategy to fabricate the bismuth nanosheets/N‑doped carbon nanoleaves (BiNS/NCL) electrode for the CDI dechlorination process. The designed architecture comprises ultrathin bismuth nanosheets intimately integrated with a leaf‐like nitrogen‐doped carbon scaffold, which is derived from a 2D bimetallic zeolitic imidazolate framework (ZIF‑L, containing Cu^2+^ and Zn^2+^ ions). Initially, the ZIF‑L nanoleaves are thermally converted into Cu nanoparticles‐embedded N‑doped carbon nanoleaves (CuNP/NCL) through controlled pyrolysis, which then serve as a conductive scaffold for the in situ growth of Bi nanosheets through galvanic replacement. The resulting 2D‑on‑2D heterointerface creates an electronic‑gradient‑driven interface, where work‑function‑mediated charge redistribution establishes a built‑in electric field directed from Bi to N‑doped carbon. Through ex situ and in situ structural/spectroscopic analyses, along with density‑functional‑theory (DFT) calculations, we elucidate how this field cooperatively modulates chloride electrosorption thermodynamics and ion‐transport kinetics, and stabilizes the reversible Bi/BiOCl phase transformation. This study not only demonstrates a high‑capacity chloride‑capture electrode but also provides a generalizable materials‑design principle in which interfacial electronic engineering and nanostructural confinement are synergistically leveraged to overcome classical trade‑offs in Faradaic deionization systems.

## 2 Results and Discussion

**FIGURE 1:**
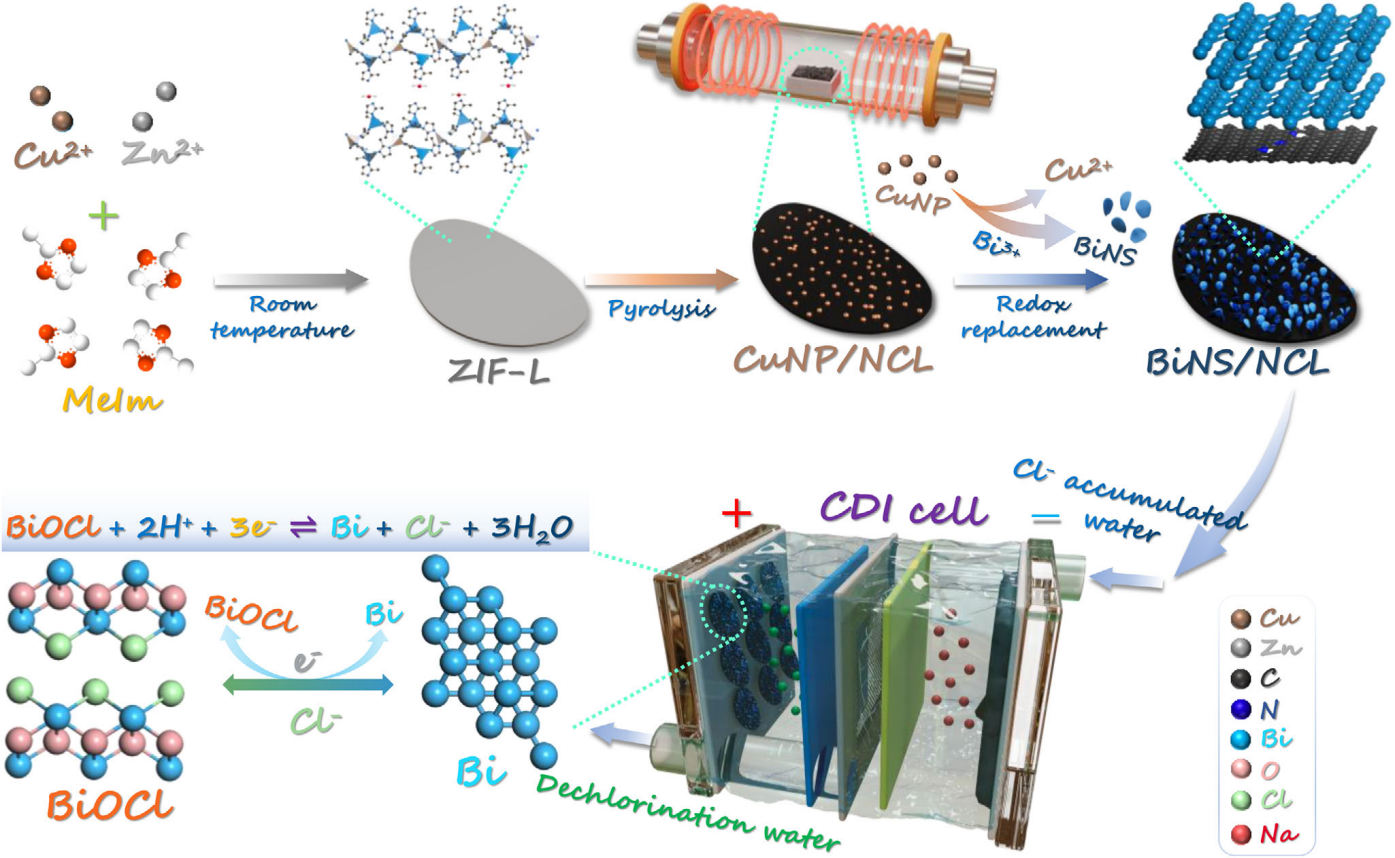
Schematic illustration of the synthesis process of the BiNS/NCL heterointerface and the reversible phase transformation between metallic Bi and BiOCl phase during the CDI dechlorination/regeneration cycle.

The BiNS/NCL nanoarchitecture was constructed through a MOF‐templated 2D‐on‐2D heterointerface engineering strategy, which integrates 2D bismuth nanosheets onto 2D N‐doped carbon nanoleaves, as schematically illustrated in Figure 1. Specifically, the ZIF‐L nanoleaves with well‐defined 2D leaf‐like morphology were first synthesized through a room‐temperature coordination reaction in an aqueous medium, with dual metal ions (Zn^2+^ and Cu^2+^) and 2‐methylimidazole (MeIm) as organic ligands [13, 34]. The as‐prepared ZIF‐L precursors were subsequently subjected to a controlled pyrolysis under a nitrogen atmosphere, leading to the formation of CuNP/NCL. During this thermal treatment, copper ions originating from the ZIF‐L precursors were reduced to metallic Cu nanoparticles, which remained spatially confined within the porous carbon architecture derived from ZIF‐L. Following this, a straightforward yet efficient galvanic replacement reaction was carried out by immersing the CuNP/NCL in a BiCl_3_ solution. Driven by the redox reaction between Bi^3+^ and Cu (2Bi^3+^ + 3Cu → 2Bi + 3Cu^2+^) [22, 35], Bi nanosheets were in situ assembled on the NCL surface, ultimately yielding the integrated BiNS/NCL heterointerface. Further details regarding the synthesis reagents and experimental procedures are provided in Sections S1.1–S1.5. The reversible phase transformations between metallic Bi and BiOCl during the CDI dechlorination/regeneration cycle are also illustrated in Figure 1. During the dechlorination step, the metallic Bi phase undergoes oxidation to form the BiOCl, while the BiOCl phase is efficiently reduced back to the metallic Bi phase during the regeneration process. Such reversible phase transformation highlights the structural robustness of the heterointerface and also demonstrates its significant potential for selective Cl^−^ capture in practical CDI applications.

**FIGURE 2:**
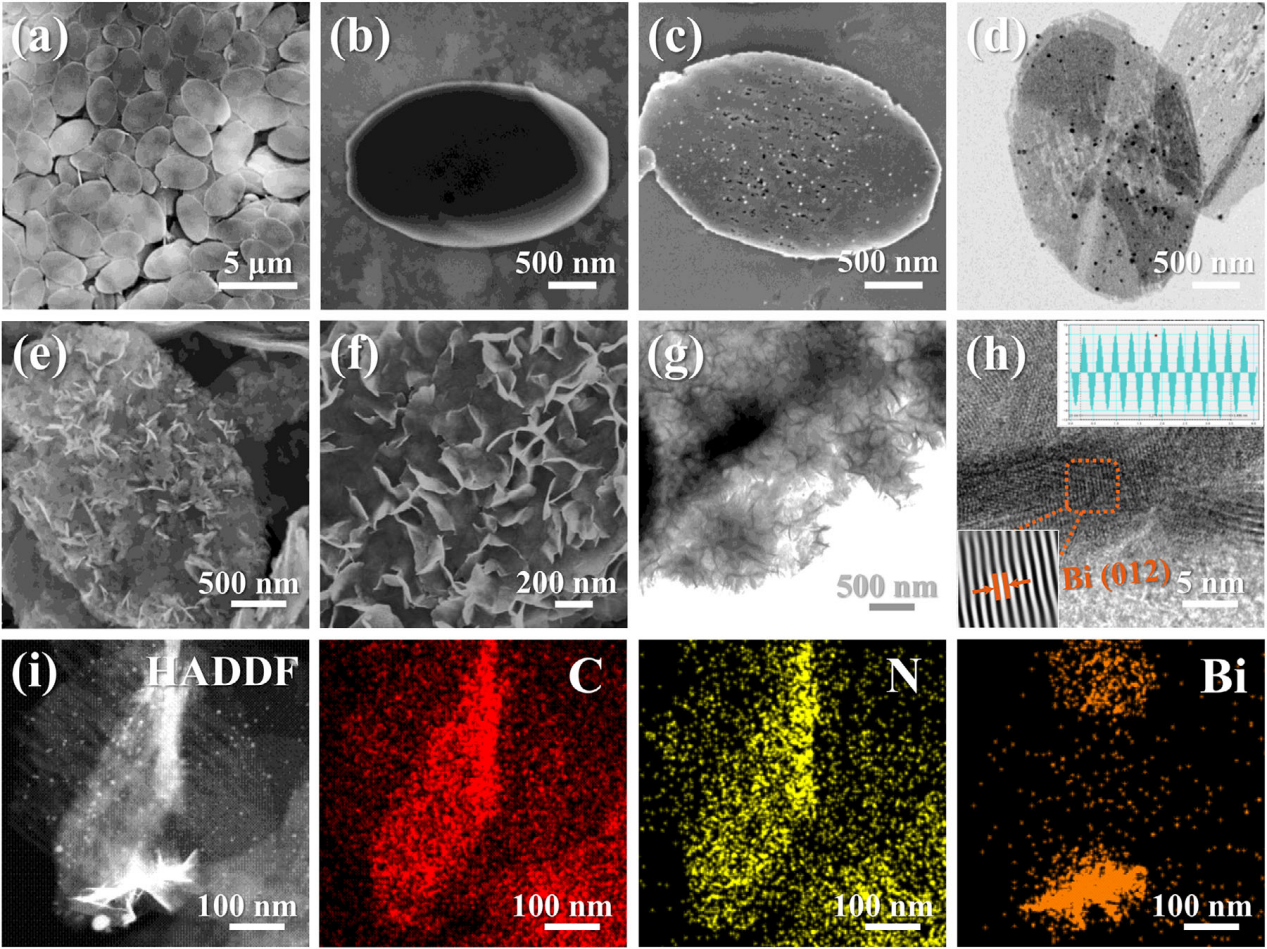
SEM images of (a,b) ZIF‐L. (c) SEM and (d) TEM images of CuNP/NCL. (e,f) SEM, (g) TEM, and (h) HRTEM images of BiNS/NCL (inset: the inverse fast Fourier transformation image and corresponding line scan of BiNS/NCL for Bi (012) plane). (i) HAADF‐STEM and corresponding EDX elemental mapping images of BiNS/NCL.

The structural and morphological evolutions from ZIF‐L precursors to CuNP/NCL and ultimately to the BiNS/NCL heterointerface were systematically investigated using scanning electron microscopy (SEM) and transmission electron microscopy (TEM). As shown in Figure 2a,b, the as‐synthesized ZIF‐L precursors exhibit a leaf‐like architecture, composed of uniformly dispersed nanoleaves with an average lateral dimension of approximately 3 µm. Upon thermal pyrolysis, the resulting CuNP/NCL largely maintains this inherent leaf‐like structure, while undergoing significant surface reconstruction, characterized by the homogeneous dispersion of copper nanoparticles within a porous N‐doped carbon matrix (Figure 2c,d). This morphological transformation might be primarily ascribed to the in situ reduction of Cu^2+^ ions, coupled with progressive carbonization of the organic ligands from the ZIF‐L frameworks. High‐resolution TEM (HRTEM) analysis (Figure S1) further confirms the presence of metallic Cu in CuNP/NCL, as evidenced by distinct lattice fringes with an interplanar spacing of approximately 0.209 nm, consistent with the (111) plane of face‐centered cubic (*fcc*) copper (JCPDS No. 04–0836) [36]. Following the galvanic replacement reaction, the original Cu nanoparticles were successfully substituted by bismuth nanosheets. The SEM image in Figure 2e illustrates the uniform assembly of Bi nanosheets on the leaf‐like NCL structure. Further SEM and TEM images in Figure 2f,g highlight the characteristic wrinkled and folded morphology of the Bi nanosheets. HRTEM analysis (Figure 2h) identifies clear lattice fringes with a spacing of ∼0.327 nm, corresponding to the (012) plane of bismuth [22, 37]. Moreover, the high‐angle annular dark‐field scanning TEM (HAADF‐STEM) images, along with the corresponding elemental mapping (Figure 2i), demonstrate the homogeneous dispersion of C, N, and Bi elements throughout the BiNS/NCL heterointerface.

**FIGURE 3:**
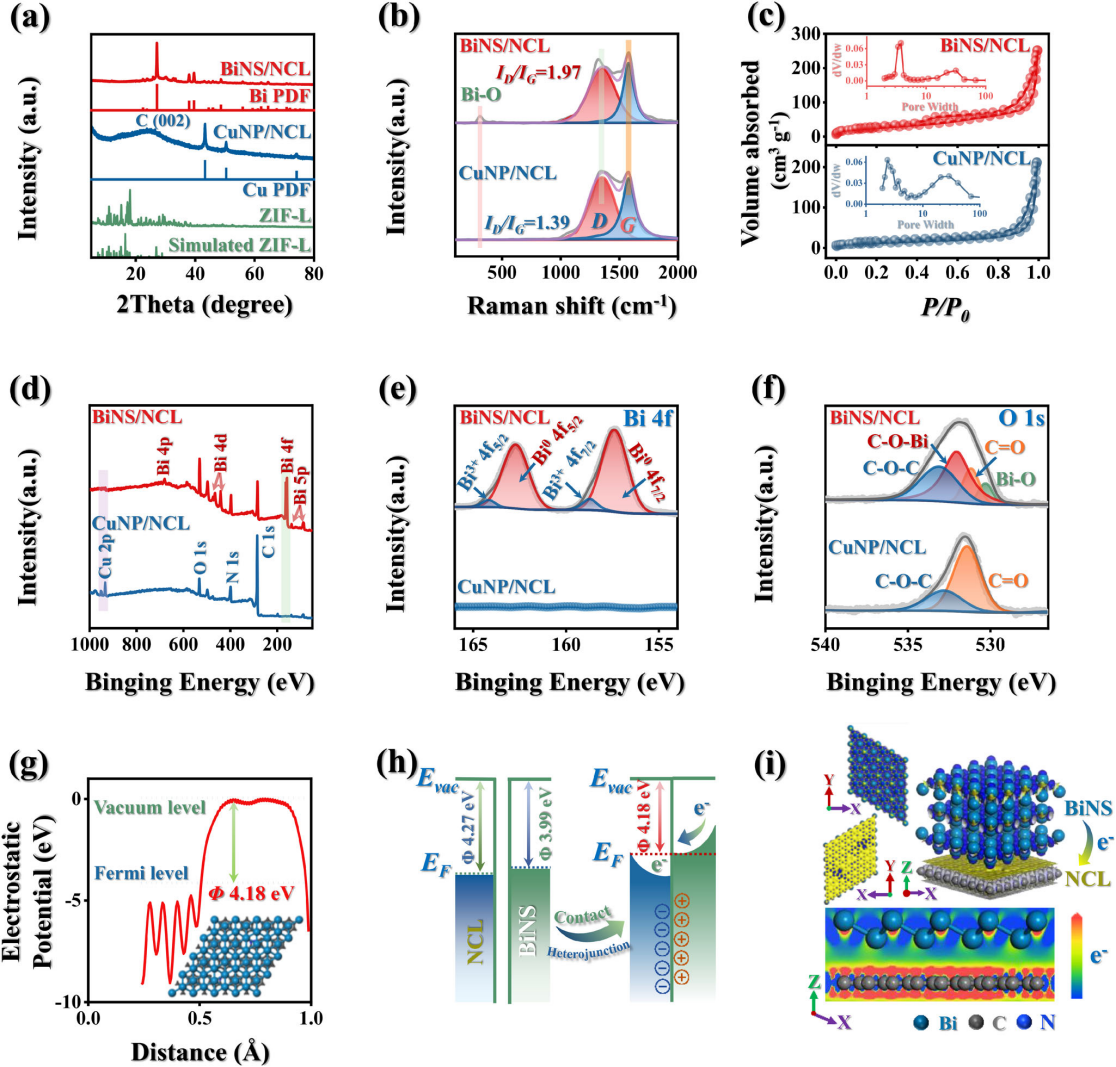
(a) XRD patterns of the ZIF‐L precursor, simulated ZIF‐L, CuNP/NCL, BiNS/NCL, together with the reference patterns of Cu PDF#04‐0836, and Bi PDF#44‐1246. (b) Raman spectra of CuNP/NCL and BiNS/NCL. (c) N_2_ adsorption‐desorption isotherms and corresponding pore size distributions of CuNP/NCL and BiNS/NCL. (d) XPS survey spectra, (e) high‐resolution Bi 4f XPS spectra, and (f) high‐resolution O 1s XPS spectra of CuNP/NCL and BiNS/NCL. (g) Electrostatic potential distribution across the BiNS/NCL interface. (h) Schematic illustration of energy‐level alignment before and after interfacial contact between BiNS and NCL. (i) Differential charge‐density distribution of BiNS/NCL along different directions and the corresponding 2D cross‐section at the BiNS/NCL interface, where charge accumulation and depletion are indicated in yellow and green, respectively.

The structural evolution from the ZIF‐L precursors to the BiNS/NCL heterointerface was systematically characterized through powder X‐ray diffraction (PXRD) analysis. As illustrated in Figure 3a, the as‐synthesized ZIF‐L precursor exhibits a well‐defined crystalline architecture, with all major diffraction peaks matching well the simulated ZIF‐L pattern [13, 34, 38]. After thermal carbonization, the characteristic ZIF‐L peaks disappear completely, accompanied by the emergence of two diffraction peaks at ∼43.3° and 50.4°. These reflections are indexed to the (111) and (200) planes of face‐centered cubic Cu (JCPDS No. 04–0836) [39, 40], suggesting the reduction of Cu^2+^ to metallic Cu^0^ nanoparticles, which are embedded within the ZIF‐L‐derived NC lattice. The subsequent galvanic replacement reaction induces a pronounced phase transformation, as evidenced by the disappearance of Cu diffraction signatures and the simultaneous appearance of new reflections at approximately 27.2°, 38.0°, and 39.6°. These are assigned to the (012), (104), and (110) crystallographic planes of rhombohedral bismuth (JCPDS No. 44–1246) [41, 42], respectively. The complementary disappearance of Cu and crystallization of Bi phases conclusively demonstrate the effective galvanic replacement process of transforming Cu nanoparticles into well‐defined Bi nanosheets, ultimately yielding the hierarchical BiNS/NCL heterointerface. Raman spectra of both CuNP/NCL and BiNS/NCL in Figure 3b show two characteristic bands at approximately 1350 cm^−1^ (*D* band) and ∼1580 cm^−1^ (*G* band), corresponding to the A_1_g mode of disordered sp^3^ carbon and the E_2_g mode of graphitic sp^2^ domains [14], respectively. Notably, the intensity ratio of *I_D_/I_G_* increases from 1.39 for CuNP/NCL to 1.97 for BiNS/NCL, indicating an enhanced degree of structural disorder or defect following the galvanic replacement reaction. This increase might be primarily attributed to the replacement of CuNP by BiNS during the redox reaction, which leaves behind vacancies and voids within the carbon structure (Figure 2c), accompanied by mild oxidative etching of the carbon surface [43]. This enhancement in disorder aligns well with the above XRD observations (Figure 3a), which show a pronounced attenuation of the graphitic (002) peak in BiNS/NCL relative to CuNP/NCL. Moreover, a small peak at ∼ 311 cm^−1^ appears in the Raman spectrum of BiNS/NCL, which can be assigned to the vibrational signature of Bi─O bonds [44], while no such Bi─O‐related features are observed in the Raman spectrum of CuNP/NCL.

The nitrogen adsorption‐desorption isotherms shown in Figure 3c reveal distinct textural characteristics for CuNP/NCL and BiNS/NCL, with both materials displaying hysteresis loops [45, 46, 47]. Notably, BiNS/NCL possesses a more pronounced hysteresis loop, signifying substantially developed mesoporosity after the galvanic replacement process. Quantitative Brunauer‐Emmett‐Teller (BET) analysis shows that BiNS/NCL possesses a specific surface area of 89.0 m^2^ g^−1^, more than twice that of CuNP/NCL (42.9 m^2^ g^−1^). The corresponding pore‐size distributions further corroborate the presence of hierarchical porosity in both materials, with a pore system dominated by mesopores and accompanied by a macroporous contribution (inset, Figure 3c).

Complementary static water contact‑angle measurements (Figure S2) reveal that BiNS/NCL exhibits a contact angle of 32.8°, significantly lower than those of bulk BiNS (47.6°) and CuNP/NCL (42.9°), indicating its enhanced surface hydrophilicity. To gain mechanistic insight into this behavior, DFT calculations were performed to evaluate the adsorption energies of water molecules on the respective surfaces. As summarized in Table S1, the calculated adsorption energies show that BiNS/NCL exhibits an adsorption energy of −0.48 eV, which is substantially more negative than that of bulk BiNS (−0.13 eV) and CuNP/NCL (−0.27 eV). The more negative adsorption energy for BiNS/NCL suggests favorable surface wettability [6], which facilitates electrolyte accessibility to the active interfaces and promotes efficient chloride ion transport during the CDI dechlorination process.

X‐ray photoelectron spectroscopy (XPS) analysis was conducted to characterize the surface chemical composition and electronic structure during the transformations from CuNP/NCL to BiNS/NCL (Figure 3d). The survey XPS spectrum of CuNP/NCL exhibits the expected characteristic peaks of C 1s, N 1s, and O 1s, along with distinct Cu 2p spin–orbit doublets. Corresponding high‑resolution analysis of the Cu 2p region (Figure S3a) further resolves this doublet into the Cu 2p_3/2_ and Cu 2p_1/2_ components, centered at approximately ∼932.6 and ∼952.5 eV, respectively [48, 49]. The survey spectrum of BiNS/NCL exhibits multiple Bi core‐level features, including Bi 4p, Bi 4d, Bi 5p, and Bi 4f doublets, accompanied by a pronounced attenuation of the Cu 2p features (Figure 3d). This systematic spectral evolution directly corroborates the successful substitution of Cu by Bi and the formation of BiNS/NCL. High‐resolution analysis of the Bi 4f region (Figure 3e) confirms that no Bi‐related features are present in the CuNP/NCL spectrum, while the BiNS/NCL spectrum resolves two intense doublets at ∼157.2 and ∼162.5 eV, corresponding to Bi^0^ 4f_7/2_ and Bi^0^ 4f_5/2_ states [50, 51], respectively, unequivocally verifying that metallic Bi constitutes the dominant chemical state. A secondary set of weaker sub‑peaks observed at ∼160.7 and ∼165.9 eV are assigned to the Bi^3+^ 4f_7/2_ and 4f_5/2_ states [44], as the characteristic of surface‑oxidized bismuth species (e.g., Bi─O bonds). Quantitative deconvolution of the Bi 4f peaks in Figure 3e further reveals that the intensities of the Bi‐O doublets constitute merely 8.5% and 5.4% of the respective metallic Bi^0^ 4f_7/2_ and 4f_5/2_ intensities, suggesting that surface oxidation is limited to a minor fraction. This finding aligns with a weak Bi─O vibrational signature observed in the Raman spectrum (Figure 3b), collectively substantiating that metallic Bi^0^ overwhelmingly dominates the chemical state of BiNS/NCL. The high‑resolution O 1s spectra (Figure 3f) further elucidate chemical rearrangements at the BiNS/NCL interface. The spectrum of CuNP/NCL shows two typical components for oxygen‑functionalized carbon, namely C─O─C (∼533.1 eV) and C═O (∼531.1 eV) [52]. While the O 1s profile of BiNS/NCL deconvolves into four fitted contributions: C─O─C (∼533.1 eV), C─O─Bi (∼532.0 eV), C═O (∼531.1 eV), and Bi─O (∼530.3 eV) [44], respectively. The emergence of distinct C─O─Bi and Bi─O components, along with a relative decrease in the C═O peak, indicates a substantive reorganization of oxygen‐containing surface species upon Bi introduction. These spectral changes suggest that interfacial reactions during the Bi‑for‑Cu replacement promote the formation of covalent or coordinative Bi─O─C linkages [53], likely through the coordination of surface oxygen species with Bi nanosheets [44]. Additionally, the high‐resolution N 1s spectra reveal the coexistence of pyridinic‑N, pyrrolic‑N, and graphitic‑N species in both CuNP/NCL and BiNS/NCL (Figure S3b). This N‑doped configuration enhances the electrical conductivity and surface wettability of BiNS/NCL, thereby facilitating interfacial charge transfer and electrolyte accessibility during the electrochemical Cl^−^ capture process.

The above XPS analysis verifies the successful formation of the BiNS/NCL heterointerface and discloses distinct interfacial bonding characteristics, suggesting pronounced electronic coupling at the interface. The simultaneous presence of metallic Bi^0^ states and C─O─Bi interfacial bonds suggests established pathways for continuous intercomponent electron transfer, underscoring how interfacial electronic structure governs charge distribution and transport dynamics. To quantitatively unravel the origin of these interfacial effects, we conducted first‐principles work function analysis. The calculated work functions are 3.99 eV for bulk BiNS, 4.27 eV for NCL, and 4.18 eV for BiNS/NCL (Figure 3g; Figure S4), respectively. This potential gradient drives spontaneous electron transfer from BiNS to NCL upon contact, resulting in Fermi level equilibration at the interface (Figure 3h). The resultant interfacial charge redistribution is explicitly visualized through the charge‐density difference mapping (Figure 3i), which reveals pronounced electron accumulation in the NCL region while concomitant depletion occurs around BiNS, indicating the formation of an interfacial dipole. This asymmetric charge configuration establishes a built‐in electric field oriented from BiNS toward NCL, which not only strengthens interfacial coupling but also induces an electron‐deficient state in BiNS. The tailored electronic structure significantly enhances the electrosorption capability of BiNS toward negatively charged chloride ions, thermodynamically favoring the nucleation of BiOCl phase during Cl^−^ capture process. The synergistic interplay between the oriented built‐in field and modulated surface electronic states collectively enhances chloride capture efficiency and BiNS/NCL stability, providing a mechanistic foundation for the superior performance of the BiNS/NCL heterointerface.

**FIGURE 4:**
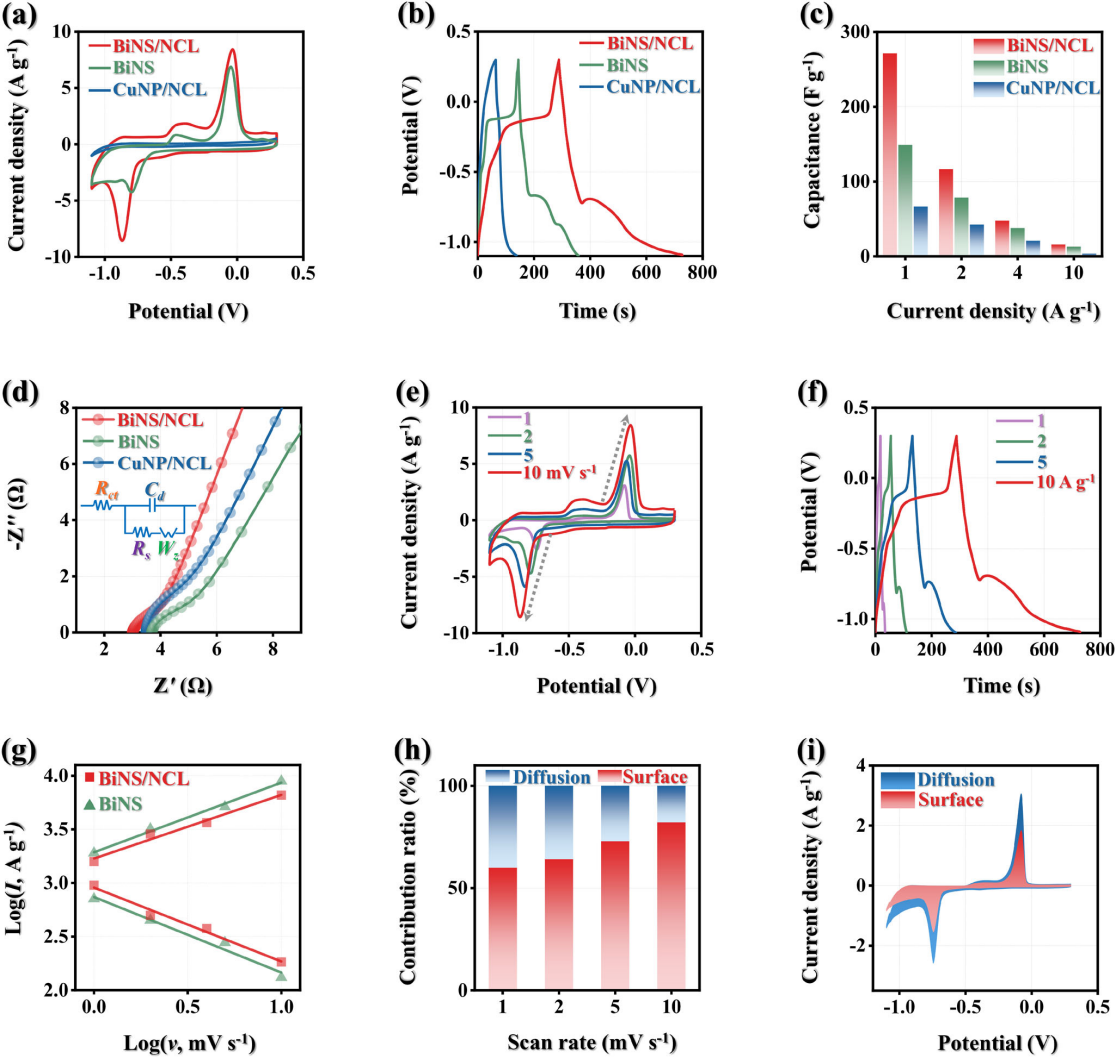
(a) CV curves at a scan rate of 10 mV s^−1^, (b) GCD profiles at a current density of 1 A g^−1^, and (c) specific capacitance values at different current densities for CuNP/NCL, bulk BiNS, and BiNS/NCL. (d) Nyquist plots of the three electrodes (inset: the equivalent circuit diagram of BiNS/NCL). (e) CV curves of BiNS/NCL at scan rates from 1 to 10 mV s^−1^. (f) GCD curves of BiNS/NCL at current densities from 1 to 10 A g^−1^. (g) The fitted curves between log (*I*, current density) and log (*v*, scan rate) in the charge and discharge processes for BiNS and BiNS/NCL. (h) Normalized proportions of surface‐ and diffusion‐controlled contributions at different scan rates for BiNS/NCL. (i) Decoupling of surface‐ and diffusion‐controlled contributions for BiNS/NCL at a scan rate of 1 mV s^−1^.

The precisely engineered interfacial electronic configuration and built‐in electric field characterized above are anticipated to fundamentally modulate electrochemical Cl^−^ storage behaviors. To experimentally verify this premise, we systematically evaluated the Cl^−^ storage performance through a series of electrochemical measurements comprising cyclic voltammetry (CV), galvanostatic charge/discharge (GCD), and electrochemical impedance spectroscopy (EIS) in a three‐electrode configuration using 1.0 mol L^−1^ NaCl electrolyte. For comparative analysis, bulk BiNS (Figure S5) and CuNP/NCL (Figure 2c) were also synthesized and examined under identical conditions (S1.4 and S1.5). As manifested in Figure 4a, both BiNS/NCL and bulk BiNS electrodes exhibit well‐defined redox couples centered at approximately 0 and −0.8 V (vs. Ag/AgCl), corresponding to the reversible phase transformation between metallic Bi and BiOCl during the dechlorination/regeneration cycle [15, 54]. In contrast, the CV curve of CuNP/NCL displays a quasi‐rectangular shape without discernible redox peaks, characteristic of electric double‐layer capacitance [14]. Remarkably, the integrated CV area of BiNS/NCL substantially surpasses that of bulk BiNS and CuNP/NCL, reflecting a markedly enhanced electrochemical Cl^−^ storage capability (Figure 4a). Complementary GCD measurements performed at 1.0 A g^−1^ within the optimized potential window (−1.1 to 0.3 V vs. Ag/AgCl) reinforce these observations (Figure 4b). The pronounced potential plateaus observed in both BiNS/NCL and bulk BiNS confirm their battery‐type Faradaic behaviors, governed by reversible redox transformations [22]. However, CuNP/NCL presents a symmetrical quasi‐triangular profile, consistent with the electric double‐layer capacitive mechanism identified in the corresponding CV analysis [55, 56]. Notably, BiNS/NCL achieves significantly extended charge/discharge durations relative to both bulk BiNS and CuNP/NCL, unambiguously reflecting its superior Cl^−^ storage capability. The specific capacitances of CuNP/NCL, bulk BiNS, and BiNS/NCL at different current densities were quantitatively calculated using Equation S1 (Section S1.6). As summarized in Figure 4c, BiNS/NCL delivers a specific capacitance of 271.2 F g^−1^ at a current density of 1 A g^−1^, substantially exceeding that of bulk BiNS (148.9 F g^−1^) and CuNP/NCL (66.7 F g^−1^), respectively.

EIS was employed to elucidate the charge transport characteristics and electrochemical conductivities of CuNP/NCL, bulk BiNS, and BiNS/NCL electrodes, with the corresponding Nyquist plots presented in Figure 4d. The experimental data, analyzed through the fitted equivalent circuit model (inset of Figure 4d), reveal that BiNS/NCL exhibits a significantly lower charge transfer resistance (*R_ct_*, 1.59 Ω) compared to bulk BiNS (*R_ct_*, 2.14 Ω) and CuNP/NCL (2.17 Ω), which indicates an improved interfacial charge transfer kinetics for BiNS/NCL [15]. Additionally, the Nyquist plot of BiNS/NCL displays a steeper slope in the low‐frequency region, which further corroborates the enhanced ion diffusion kinetics and efficient charge transport within the BiNS/NCL. To further assess the electrochemical behavior, CV measurements were conducted at scan rates ranging from 1 to 10 mV s^−1^ for CuNP/NCL, bulk BiNS, and BiNS/NCL electrodes (Figure 4e; Figure S6). All three electrodes largely maintain their CV profiles as the scan rate increases, indicating their robust structural integrity and electrochemical reversibility. However, both bulk BiNS and BiNS/NCL exhibit a progressive broadening of redox peaks and an increasing separation between reduction and oxidation potentials at higher scan rates. This potential polarization phenomenon originates from the competing effects between charge transfer kinetics and mass transport limitations, where accelerated scanning conditions intensify diffusion resistance and induce kinetic hysteresis in the Faradaic processes [57]. Further insights were gained from GCD profiles, presented in Figure 4f and Figure S7, which were recorded over a current density range of 1–10 A g^−1^. Both bulk BiNS and BiNS/NCL electrodes maintain stable potential plateaus across this extended range, indicating the reversibility of their Faradaic charge storage mechanisms under varying rate conditions. While CuNP/NCL retains its characteristic quasi‐triangular charge–discharge profile without the distinct plateaus, indicating capacitive charge storage characteristics. In contrast, BiNS/NCL exhibits standout performance, consistently delivering the most well‐defined voltage plateaus and longest discharge durations across the entire current density range tested, surpassing both CuNP/NCL and bulk BiNS.

To investigate the electrochemical kinetics of CuNP/NCL, bulk BiNS, and BiNS/NCL, we systematically evaluated their charge storage mechanisms and quantitatively deconvoluted the contributions from surface‑controlled (capacitive) and diffusion‑controlled (Faradaic) processes. The relationship between scan rate (*v*, mV s^−1^) and peak current (*I*, A g^−1^) was calculated using Equations S2 and S3 (Section S1.6). As depicted in Figure 4g, the linear fitting of log(*I*) versus log(*v*) yields straight lines during both charge and discharge cycles. The corresponding *b*‐values for BiNS/NCL and bulk BiNS are approximately 0.68 and 0.63, respectively, indicating that their capacitive contributions are jointly governed by surface‐controlled and diffusion‐controlled processes [15]. The contributions from surface‐ and diffusion‐controlled processes were further quantified using current‑deconvolution analysis based on Equation S4 (Section S1.6). As shown in Figure 4h, the capacitive contribution of BiNS/NCL increases from 59.9% to 82.2% as the scan rate increases from 1 to 10 mV s^−1^, highlighting the dominant role of surface‑controlled kinetics under higher scan rates. At a lower scan rate of 1 mV s^−1^, BiNS/NCL possesses a considerable diffusion‑controlled contribution of 40.1 % (Figure 4i), whereas bulk BiNS exhibits a higher diffusion contribution of 57.8 % (Figure S8). This shift in the relative contributions of diffusion‐controlled and surface‐controlled processes in BiNS/NCL reflects its superior electrochemical performance, particularly in facilitating rapid charge storage at higher scan rates.

**FIGURE 5:**
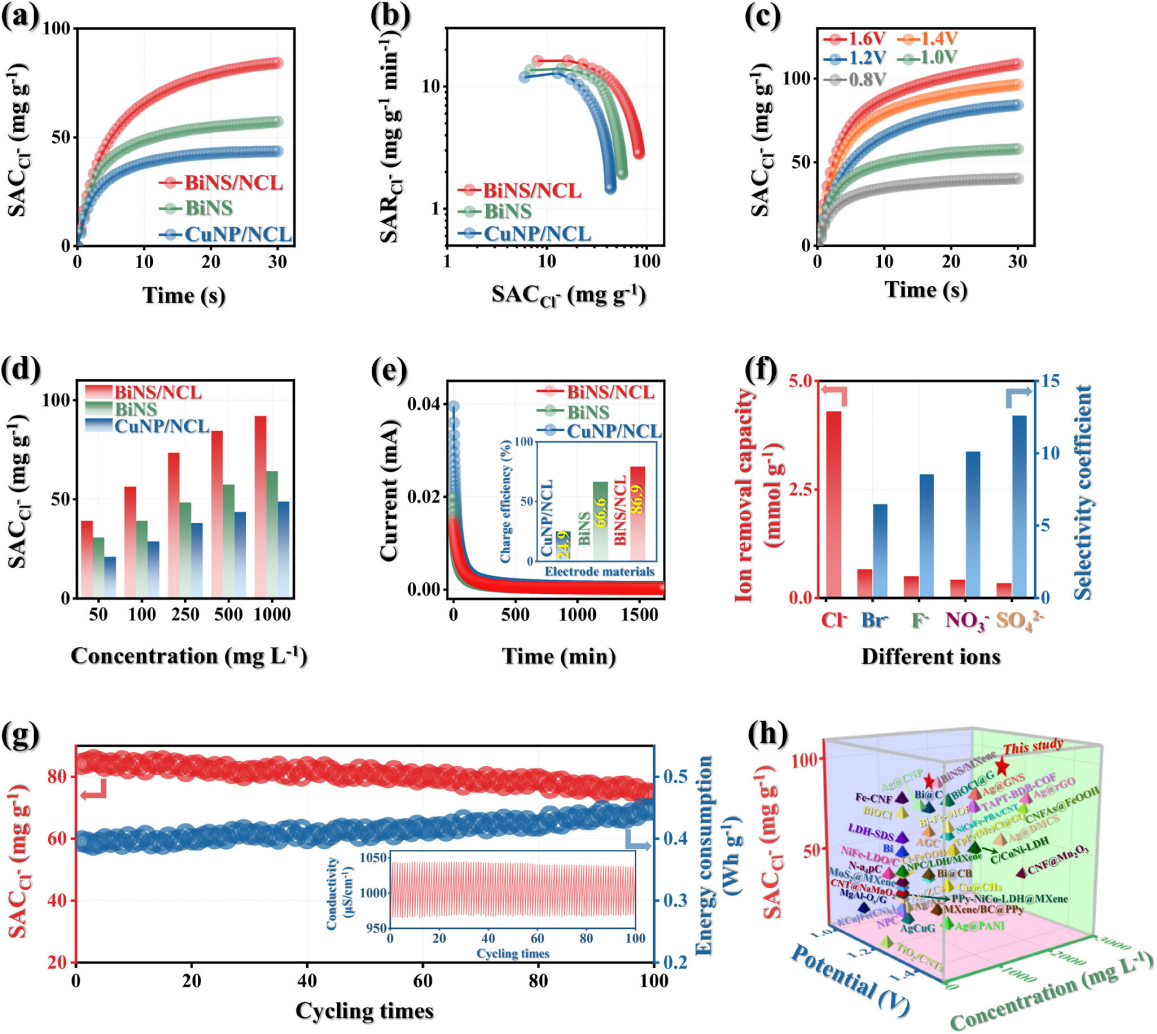
(a) Time‐dependent SAC_Cl‐_ profiles and (b) corresponding CDI Ragone plots for CuNP/NCL, BiNS, and BiNS/NCL measured in 500 mg L^−1^ NaCl solution at 1.2 V. (c) Dynamic SAC_Cl‐_ evolution of BiNS/NCL in 500 mg L^−1^ NaCl solution under applied voltages from 0.8 to 1.6 V. (d) SAC_Cl‐_ values for CuNP/NCL, BiNS, and BiNS/NCL at NaCl concentrations ranging from 50 to 1000 mg L^−1^. (e) Current‑time responses in 500 mg L^−1^ NaCl at 1.2 V (inset: corresponding charge efficiencies). (f) Cl^−^ removal performance of BiNS/NCL in a 10 mmol L^−1^ mixed‑ion solution at 1.2 V (red bars) and the corresponding selectivity coefficients for Cl^−^ over Br^−^, F^−^, NO_3_^−^, and SO_4_^2−^ (gray‑blue bars). (g) Cycling stability and energy consumption of BiNS/NCL over 100 consecutive dechlorination/regeneration cycles (inset: real‑time conductivity profile during operation) measured in 500 mg L^−1^ NaCl solution (Volume, 32 mL) at 1.2 V and 25 °C, with each cycle consisting of 30 min for both adsorption and desorption phases using an electrode loaded with 16 mg of BiNS/NCL. (h) Comparison of the Cl^−^ capture capacity for BiNS/NCL with previously reported Faradaic dechlorination electrodes.

The chloride‐ion capture performance of BiNS/NCL was systematically assessed in a hybrid CDI (HCDI) configuration, wherein BiNS/NCL operated as the Faradaic anode and commercial activated carbon (AC) functioned as the capacitive cathode (assembly details provided in Section S1.7). During the dechlorination process, Cl^−^ ions are selectively driven toward the BiNS/NCL anode, where metallic Bi undergoes a reversible Faradaic transformation into BiOCl phase. Upon regeneration, the BiOCl phase is reduced back to the metallic Bi phase, thereby enabling a highly reversible dechlorination/regeneration cycle (see schematic in Figure 1). To benchmark the intrinsic Cl^−^ capture capability, control experiments were performed under identical operational conditions using CuNP/NCL and bulk BiNS as reference anodes. The specific adsorption capacity for Cl^−^ (SAC_Cl‐_) was quantified by monitoring the real‑time Cl^−^ concentration during the dechlorination process according to Equation S5 (Section S1.7). The time‑dependent SAC_Cl‐_ profiles for CuNP/NCL, BiNS, and BiNS/NCL were measured at an initial Cl^−^ concentration of 500 mg L^−1^ under an applied voltage of 1.2 V and are shown in Figure 5a. All electrodes display a rapid increase in SAC_Cl‐_ values during the initial stage of dechlorination, followed by progressively slower Cl^−^ uptake as the system approaches equilibrium. Remarkably, BiNS/NCL delivers a maximum SAC_Cl‐_ value of 84.2 mg g^−1^, substantially exceeding those of CuNP/NCL (43.6 mg g^−1^) and bulk BiNS (57.3 mg g^−1^), which highlights its superior performance in chloride capture. This dechlorination performance is further supported by the Ragone analysis (Figure 5b), where BiNS/NCL is positioned in the optimal upper‐right region, reflecting its balanced excellence in both SAC_Cl‐_ and rapid Cl^−^ adsorption rate (SAR_Cl‐_). The maximum SAR_Cl‐_ of BiNS/NCL, calculated using Equation S6 in Section S1.7, reaches an impressive value of 16.2 mg g^−1^ min^−1^, further attesting to its rapid chloride capture capability.

The effect of applied voltages and initial Cl^−^ concentrations on dechlorination performance was systematically investigated. As depicted in Figure 5c and Figure S9, the SAC_Cl‐_ values of BiNS/NCL, BiNS, and CuNP/NCL electrodes increase markedly as the applied voltage rises from 0.8 to 1.6 V, suggesting the enhanced Cl^−^ migration driven by the electric field and facilitated by interfacial electrosorption processes [6, 15]. Across the entire voltage range, BiNS/NCL consistently outperforms the other materials, achieving an impressive SAC_Cl‐_ of 108.7 mg g^−1^ (Figure 5c). These results are further substantiated by Ragone plot analysis (Figure S10), in which BiNS/NCL consistently occupies the upper‐right region, clearly outperforming both BiNS and CuNP/NCL. Notably, a high SAR_Cl‐_ of 24.8 mg g^−1^ min^−1^ for BiNS/NCL is achieved, underscoring its superior capacity‐rate balance under varying voltage conditions (Figure S10). In addition, as illustrated in Figure 5d, the SAC_Cl_^−^ values for all electrodes show a monotonic increase with rising NaCl concentrations. Within the concentration range of 50–1000 mg L^−1^, BiNS/NCL consistently maintains the highest dechlorination capacity, reaching a maximum SAC_Cl‐_ of 91.9 mg g^−1^ at 1000 mg L^−1^, which surpasses those of both BiNS and CuNP/NCL under identical conditions (Figure S11a–c). Notably, the rate of increase gradually slows at higher concentrations, reflecting a saturation behavior that aligns with Langmuir‐type adsorption [58], where the active sites become progressively occupied as Cl^−^ concentration increases. This trend is further governed by the concentration‑dependent driving force for the reversible Bi/BiOCl phase transformation, alongside kinetic constraints associated with solid‑state diffusion and interfacial charge transfer during Cl^−^ capture. The corresponding Ragone plot further reinforces this advantage, with BiNS/NCL again occupying a more favorable upper‐right position, indicative of its robust capacity‐rate characteristics across a wide concentration window (Figure S11d–f). Furthermore, the Langmuir isotherm model was applied to fit the experimental SAC_Cl‐_ values of BiNS/NCL at Cl^−^ concentrations ranging from 50 to 1000 mg L^−1^ under an applied voltage of 1.2 V. As shown in Figure S12 and Table S2, the fitted model yields a theoretical maximum SAC_Cl‐_ (*q_m_*) of 97.8 mg g^−1^ with a correlation coefficient of 0.997, indicating good agreement between the Langmuir model and the experimental data.

Real‐time current profiles were recorded throughout the dechlorination process, while the continuous adsorption and desorption dynamics of Cl^−^ ions by BiNS/NCL, BiNS, and CuNP/NCL electrodes were systematically monitored (Figure 5e). The charge efficiency (*Λ*), calculated according to Equation S7 (Section S1.7), serves as an indicator of energy utilization efficiency. As illustrated in the inset of Figure 5e, BiNS/NCL demonstrates a markedly higher *Λ* of 86.9%, compared to 66.6% for BiNS and 24.9% for CuNP/NCL, highlighting its significantly superior charge efficiency. Moreover, the specific energy consumption (*E*), derived from Equation S8 (Section S1.7), further reveals the energy‑efficient performance of BiNS/NCL, which achieves an exceptionally low *E* of 0.39 Wh g^−1^, lower than that of BiNS (0.57 Wh g^−1^) and CuNP/NCL (1.35 Wh g^−1^). These findings collectively emphasize the exceptional energy efficiency characteristics of BiNS/NCL, supporting its viability for low‑energy electrochemical dechlorination. Meanwhile, the Cl^−^ removal selectivity of BiNS/NCL was further assessed in a mixed‑anion solution containing equimolar concentrations (10 mmol L^−1^ each) of Br^−^, F^−^, NO_3_^−^, SO_4_^2−^, and Cl^−^ (Section S1.8). As displayed in Figure 5f (left, red bars), the Cl^−^ removal capacity on BiNS/NCL reaches 4.3 mmol g^−1^, significantly exceeding those of the coexisting anions. The corresponding Cl^−^ selectivity coefficients over Br^−^, F^−^, NO_3_^−^, and SO_4_^2−^, calculated according to Equation S9 in Section S1.8, are 6.5, 8.5, 10.1, and 12.6, respectively (right, gray‑blue bars, Figure 5f). This pronounced selectivity, maintained even in complex ionic matrices, underscores the capability of BiNS/NCL for targeted Cl^−^ capture, highlighting its promise for real‑world water‑treatment applications.

The long‐term cycling stability of the BiNS/NCL electrode was investigated in a 500 mg L^−1^ NaCl electrolyte at 1.2 V to evaluate its practical applicability, with the corresponding real‑time conductivity variations depicted in the inset of Figure 5g. Remarkably, the BiNS/NCL electrode demonstrates a minimal capacity degradation, maintaining 88.3% of its initial SAC_Cl‐_ retention after 100 consecutive dechlorination/regeneration cycles (Figure 5g, left). Concurrently, the charge efficiency remains highly stable throughout cycling, accompanied by a consistently low specific energy consumption in the range of 0.39–0.45 Wh g^−1^ (Figure 5g, right). The outstanding cycling stability originates from the nanoconfinement effects of the NCL conductive nanostructured supports, which spatially confine the Bi/BiOCl active phases, suppress aggregation of BiNSs (a common drawback of bare bismuth), and accommodate volume changes during repeated phase transitions [22]. Post‐cycling TEM and HRTEM characterizations in Figure S13 further confirm no discernible morphological changes, nanosheet agglomerations, or lattice aggregations of the BiNS after 100 dechlorination/regeneration cycles, suggesting the structural integrity of BiNS/NCL after extended cycling. The modest capacity decay observed after extended cycling might be primarily attributed to the limited irreversible phase transformation from metallic BiNS to BiOCl phase during 100 cycles. This slight transformation is supported by XRD analysis (Figure S14), which shows the co‐existence of metallic BiNS and a minor BiOCl phase in the cycled BiNS/NCL electrode. Furthermore, additional XPS analysis in Figure S15 shows that the integrated peak area ratios corresponding to metallic BiNS (Bi^0^ 4f_5/2_ and 4f_7/2_) recover to 87.0% – 87.3% of their initial intensities after 100 cycles, indicating that only a small fraction of BiNS undergoes irreversible conversion to BiOCl phase. Collectively, these results underscore the robust cycling performance, structural durability, and energy‑efficient operation of BiNS/NCL, positioning it as a highly promising candidate for long‑term dichlorination. ICP‑MS analysis further reveals an exceptionally low bismuth concentration of 1.07 µg L^−1^ in the electrolyte after 100 cycles, further confirming negligible dissolution of Bi species and underscoring the structural robustness of the BiNS/NCL heterointerface. Furthermore, in comparison with state‑of‑the‑art faradaic dechlorination electrodes (Table S3; Figure 5h), BiNS/NCL shows competitive or superior performance relative to these electrodes, delivering outstanding dechlorination capacity and superior SAC_Cl‐_ values across a broad range of NaCl concentrations (250–3000 mg L^−1^) and applied voltages of 1.2–1.6 V.

**FIGURE 6:**
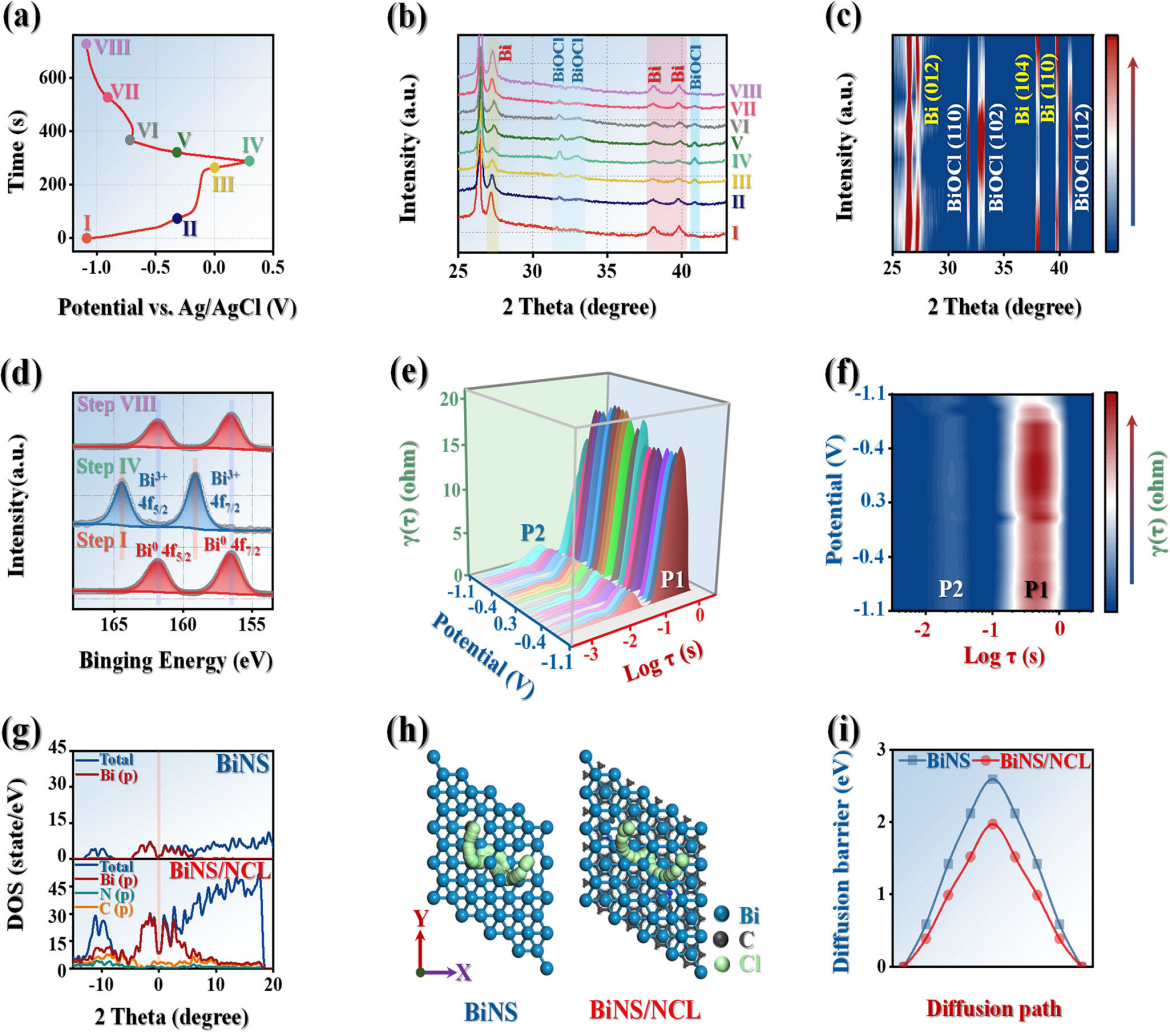
Operando structural and electronic evolution of the BiNS/NCL electrode during charge–discharge cycling. (a) Schematic of one complete charge–discharge cycle (Steps I —VIII) for the BiNS/NCL electrode. (b) Ex situ XRD patterns and (c) corresponding contour plots acquired at different charging/discharging stages. (d) High‑resolution Bi 4f XPS spectra collected at the initial (Step I), fully charged (Step IV), and discharged (Step  VIII) states. (e) Distribution of relaxation times (DRT) derived from in situ EIS at selected potentials, and (f) DRT contour map over the full charging‐discharging process. (g) Total and partial density of states (DOS) for bulk BiNS and BiNS/NCL. (h) Schematic of chloride diffusion pathways on bulk BiNS and BiNS/NCL surfaces. (i) Corresponding CI‑NEB‑calculated diffusion energy barriers for Cl^−^ on bulk BiNS and BiNS/NCL.

To elucidate the possible Cl^−^ capture mechanism, ex situ XRD and XPS analyses were performed on the BiNS/NCL electrode, enabling continuous tracking of its structural and chemical evolution throughout a full charge–discharge cycle (Figure 6a). The ex situ XRD patterns, presented in Figure 6b,c, reveal distinct and well‐defined phase transitions in BiNS/NCL during both the dechlorination (charging, Steps I to IV), and regeneration (discharging, Step IV to VIII) processes. Specifically, the diffraction peaks corresponding to the metallic Bi phase (012), (104), and (110) [59, 60] are progressively diminished from Steps I to IV, while concomitantly, the characteristic peaks of BiOCl (110), (102), and (112) [61, 62] emerge and intensify during Cl^−^ capture. In contrast, during the discharging process (Steps IV to VIII), the intensities of BiOCl peaks gradually diminish, while the metallic Bi diffraction peaks reappear, signaling a reversible conversion from BiOCl back to the metallic Bi. These XRD observations conclusively demonstrate that BiNS/NCL undergoes a reversible phase transformation between the metallic Bi phase and BiOCl during the dechlorination/regeneration cycle [63]. Additionally, ex situ XPS measurements were performed to investigate the chemical valence states of BiNS/NCL throughout the charge–discharge process. The high‐resolution Bi 4f XPS spectra in Figure 6d show that, at Step I (initial state), only the characteristic peaks of metallic Bi (Bi^0^ 4f_7/2_ and Bi^0^ 4f_5/2_) are present. Upon reaching the fully charged state (Step IV), the Bi° characteristic peaks are almost entirely vanished, replaced by characteristic peaks corresponding to Bi^3+^, signifying the formation of BiOCl phase [54, 64]. During the subsequent discharging process (Step VIII), the Bi^0^ peaks nearly revert to their original intensities, reflecting the regeneration of the metallic Bi phase, which is in excellent agreement with the XRD data (Figure 6b,c). This reversible phase transition, therefore, underpins the pronounced Cl^−^ selectivity of BiNS/NCL, as BiOCl formation is uniquely driven by Cl^−^ capture, whereas competing anions lack such a phase‑transformation pathway.

In situ EIS, coupled with distribution of relaxation times (DRT) analysis [65, 66], was further conducted to examine the electrochemical conductivities of BiNS/NCL across various charge states (Figure 6e,f; Section S1.9). The DRT deconvolution reveals two distinct electrochemical processes: (I) a low‐frequency relaxation process (Peak P1, log τ = ∼−1 to 0), associated with significant resistance variations arising from the solid‐state diffusion limitations [67, 68] during the reversible phase transformation between Bi and BiOCl; and (II) a medium‐frequency relaxation process (Peak P2, log τ = −2 to −1), corresponding to potential‐dependent shifts in the interfacial charge transfer resistance [67, 68, 69] governing Cl^−^ ion migration at the BiNS/NCL electrode‐electrolyte interface. These dynamic processes exhibit characteristic potential dependencies. Specifically, during the charging process, as the voltage increases from −1.1 to ∼0.3 V, the resistance of BiNS/NCL increases owing to the formation of the resistive BiOCl phase. While the corresponding relaxation peak experiences a slight shift toward a lower log τ, further reflecting the electrochemical evolution at the electrode‐electrolyte interface during phase transformation. Conversely, during the full discharging process at approximately −1.1 V, BiNS/NCL demonstrates a pronounced decrease in resistance, corresponding to the electrochemical reduction from BiOCl to conductive metallic Bi, which restores the conductivity of BiNS/NCL. Such well‑defined and potential‑dependent relaxation processes reflect the kinetic advantages conferred by the heterointerface, in contrast to the often poorly resolved charge transfer and diffusion characteristics for bare bismuth‑based electrodes [22]. These ex situ XRD, XPS, and in situ EIS analyses collectively demonstrate that the superior Cl^−^ capture performance of BiNS/NCL arises from its reversible phase transition between metallic Bi and BiOCl. This transformation, together with enhanced charge transfer and solid‐state diffusion processes, facilitates efficient electrochemical dechlorination and regeneration, thereby endowing BiNS/NCL with a distinct kinetic advantage for Cl^−^ capture over competing anions.

DFT calculations provide an atomistic and electronic perspective on the exceptional Cl^−^ capture performance of BiNS/NCL. As previously discussed in Figure 3h,i, the pronounced work‐function difference between BiNS and NCL drives substantial interfacial charge redistribution, giving rise to a built‐in electric field oriented from BiNS toward NCL. This interfacial polarization establishes an electron‐deficient surface environment on BiNS (Figure 3i), which thermodynamically favors electrosorption [70, 71] and promotes the reversible phase transformation during cycling. Such an electronic reorganization is consistent with the experimentally observed evolution of interfacial charge‐transfer resistance in the EIS measurements (Figure 6e,f). The electronic consequences of this interfacial modulation are further elucidated by projected density of states (PDOS) analysis. As shown in Figure 6g, the Bi 6p band center in BiNS/NCL shifts upward relative to bulk BiNS, accompanied by a substantial increase in states near the Fermi level (9.31 vs. 1.46 states eV^−1^). This redistribution of electronic states enhances the Coulombic interaction with anionic species (e.g., Cl^−^) [72, 73], which provides an electronic‐structure basis for the preferential Cl^−^ electrosorption and the subsequent phase conversion observed experimentally (Figure 6b–d). Consistent with this PDOS analysis, Cl^−^ adsorption on BiNS/NCL is thermodynamically more favorable than on bulk BiNS, as evidenced by a more negative adsorption energy (−3.54 vs. −2.93 eV, Table S4) and a substantially shortened Bi─Cl bonding distance (∼2.6 vs. ∼3.8 Å), indicating stronger electronic coupling at the BiNS/NCL interface. Beyond thermodynamics, the kinetic aspects of this configuration were further explored by quantifying the chloride diffusion energy barrier using the climbing‐image nudged elastic band (CI‐NEB) method [22, 74, 75]. Although Cl^−^ diffusion follows a similar hole‐top‐hole migration pathway on both BiNS/NCL and bulk BiNS surfaces (Figure 6h; Figure S16), the BiNS/NCL exhibits a substantially lower energy barrier (1.97 eV) compared to bulk BiNS (2.59 eV, Figure 6i). This reduced barrier stems from the electronic modulation imparted by NCL, which weakens excessive Cl^−^ binding and facilitates lateral migration across the interface. Collectively, the DFT results reveal that interfacial charge redistribution simultaneously strengthens Cl^−^ electrosorption thermodynamics and lowers surface diffusion barriers, effectively coupling ion transport with the reversible phase transformation, while such advantages are inherently absent in bulk bismuth. This synergistic electronic‑structural effect endows BiNS/NCL with a pronounced intrinsic affinity for Cl^−^, providing a mechanistic basis for its high chloride capture performance and selectivity observed in complex ionic environments.

## 3 Conclusion

This study presents an effective strategy for chloride capture in CDI through the strategic design of a MOF‐derived BiNS/NCL heterointerface. The engineered 2D‑on‑2D heterointerface between bismuth nanosheets and nitrogen‑doped carbon nanoleaves creates an interfacial built‑in electric field that collectively enhances chloride adsorption thermodynamics, accelerates ion‑transport kinetics, and stabilizes the reversible Bi/BiOCl phase transformation. As a result, the BiNS/NCL electrode demonstrates remarkable chloride adsorption capacity, rapid uptake kinetics, high charge efficiency, excellent chloride selectivity, and strong cycling stability, with substantial capacity retention over extended cycling. Combined ex situ/in situ spectroscopic analyses and DFT simulations elucidate that the interfacial electric field facilitates electron transfer, lowers the chloride diffusion energy barrier, and synchronizes thermodynamic and kinetic processes. These findings provide a coherent mechanistic understanding of interface‑governed electrochemical dechlorination and establish a generalizable heterointerface‑engineering paradigm for sustainable and energy‐efficient electrochemical water treatment technologies.

## Conflicts of Interest

The authors declare no conflicts of interest.

## Supporting information




**Supporting File**: advs75448‐sup‐0001‐SuppMat.docx.

## Data Availability

The data that support the findings of this study are available from the corresponding author upon reasonable request.
