# Supplementary material for: Heterointerface Engineering of Bismuth Nanosheets/Nitrogen‐Doped Carbon Nanoleaves Enables High‑Performance Electrochemical Dechlorination
Source: Adv Sci (Weinh). 2026 Apr 29;13(41):e75448. doi: 10.1002/advs.75448 (PMC13335483; doi:10.1002/advs.75448)
Supplement: Supplementary file 1 — Supporting File: advs75448‐sup‐0001‐SuppMat.docx. [file ADVS-13-e75448-s001.docx]

**S1. Experimental Section**

***S1.1. Chemicals and*** ***Reagents***

Copper (II) nitrate trihydrate (Cu(NO_3_)_2_·3H_2_O, ≥99.0%), zinc nitrate hexahydrate (Zn(NO_3_)_2_·6H_2_O, ≥99.0%), and 2-methylimidazole (MeIm, 98%) were purchased from Macklin Biochemical Reagent (Shanghai, China). Sodium chloride (NaCl, ≥99.5%), dimethyl sulfoxide (DMSO, ≥99.0%), ethylene glycol (99%), and bismuth trichloride (BiCl_3_, ≥99.9%) were obtained from Aladdin Scientific Reagent (Shanghai, China). Sodium borohydride (NaBH_4_, 98%), Polyvinylidene fluoride (PVDF 6020), carbon black (Super P), N‑methylpyrrolidone (NMP, 99.5%), and Nafion solution (5*wt.*%) were provided by Sigma-Aldrich (Merck, Shanghai, China). All chemicals were used as received without further purification.

***S1.2. Characterizations***

The morphology and microstructure of the prepared materials were examined by field‑emission scanning electron microscopy (FE‑SEM, Hitachi SU8600, Japan) at an acceleration voltage of 10.0 kV. Transmission electron microscopy (TEM), high-resolution TEM (HRTEM), and energy‑dispersive X‑ray spectroscopy (EDS) elemental mapping were conducted on a JEM‑2100F instrument (JEOL, Japan) operated at 200 kV. Wide-angle X-ray diffraction (XRD) patterns were acquired on a Rigaku SmartLab SE Automated Multipurpose X-ray Diffractometer with monochromatic Cu Kα radiation (40 kV, 40 mA) at a scan rate of 2 ° min^-1^ over a 2*θ* range of 5 - 80°. Surface elemental composition and chemical states were analyzed by X-ray photoelectron spectroscopy (XPS) on a PHI Quantera SXM (ULVAC-PHI) system with monochromatic Al *Kα* radiation; and all binding energies were referenced to the adventitious carbon C 1s peak at 284.5 eV. Nitrogen adsorption-desorption isotherms were recorded at 77 K using an Autosorb‑iQ analyzer (Quantachrome, USA), and specific surface areas were derived from the Brunauer-Emmett-Teller (BET) model, while pore‑size distributions were obtained via the Barrett-Joyner-Halenda (BJH) method. Surface wettability was evaluated by static water contact angle measurements performed via the sessile‑drop method at 30 ± 2 °C on a JY-PHB contact angle instrument (Jinhe, Chengde, China). During the CDI dechlorination experiments, Cl^-^ concentration (present as NaCl) was monitored in real time using a REX DDSJ‑308F conductivity meter (INESA Scientific Instrument, Shanghai, China). The concentrations of Cl^-^, Br^-^, NO_3_^-^, and SO_4_^2-^ were quantified by a high‑pressure ion chromatography (HPIC, Thermo Scientific Dionex ICS‑6000, ThermoFisher, USA). Inductively coupled plasma mass spectrometry (ICP-MS, ICAP-Q, Thermo, USA) was employed to analyze the bismuth concentration in the electrolyte after 100 cycles.

***S1.3. Synthesis of ZIF-L precursor***

ZIF-L was synthesized through a coordination‑driven self‑assembly route in aqueous medium [S1, S2]. Typically, an aqueous metal-ion precursor solution was prepared by dissolving Cu(NO_3_)_2_·3H_2_O (1 mmol, 0.242 g) and Zn(NO_3_)_2_·6H_2_O (1 mmol, 0.298 g) in 15 mL of deionized water. Separately, an organic ligand solution was obtained by dissolving MeIm (12 mmol, 0.985 g) in 20 mL of deionized water. Under continuous stirring at 800 rpm, the Cu^2+^/Zn^2+^ precursor solution was rapidly introduced into the MeIm solution. The resulting mixture was maintained under vigorous stirring (800 rpm) for 1 h, followed by reduced stirring speed (300 rpm) for an additional 8 h to promote ZIF-L crystal growth. The resulting light blue precipitate was collected by centrifugation, thoroughly washed with deionized water, and finally dried at 60 °C overnight to yield the ZIF-L precursor.

***S1.4. Synthesis of CuNP/NCL***

CuNP/NCL was synthesized via a controlled thermal pyrolysis of the ZIF‑L precursor under an inert atmosphere. In a typical procedure, 500 mg of the ZIF-L precursor was placed in an alumina boat and positioned in a horizontal tube furnace under a continuous N_2_ flow. The thermal treatment comprised two distinct stages: first, the temperature was ramped from room temperature to 350 °C at a controlled rate of 2 °C min^-1^ and held for 2 h. Subsequently, the temperature was further elevated to 800 °C at the same heating rate and maintained for 3 h to promote carbonization of the residual carbon matrix and the in situ formation of metallic copper nanoparticles via thermal reduction of the copper species in ZIF‑L [S3]. After natural cooling to room temperature, the resulting black powder was collected as the final CuNP/NCL.

***S1.5. Synthesis of BiNS/NCL and bulk BiNS***

The BiNS/NCL heterostructure was constructed via a precisely controlled galvanic replacement reaction based on the redox potential difference between metallic Cu and Bi^3+^ ions [S4, S5]. Typically, 100 mg of pre‑synthesized CuNP/NCL was dispersed in 20 mL of DMSO under ultrasonication to form a homogeneous suspension. Concurrently, a bismuth precursor solution was prepared by dissolving 1.0 g of BiCl_3_ (3.17 mmol) in 15 mL of DMSO under continuous stirring until complete dissolution. The CuNP/NCL suspension was then introduced dropwise into the BiCl_3_ solution at a controlled rate of 1 mL·min^-1^ under constant stirring (500 rpm), allowing progressive interfacial contact between the metallic CuNP from CuNP/NCL and Bi^3+^ species. The reaction mixture was subsequently heated at 70 °C for 5 h under a N_2_ atmosphere to drive the complete phase transformation. During this process, the metallic Cu nanoparticles (*φ*(Cu^2+^/Cu) = +0.340 V, *vs.* SHE) served as sacrificial templates and were oxidized to Cu^2+^ while simultaneously reducing Bi^3+^ (*φ*(Bi^3+^/Bi) = +0.308 V, *vs.* SHE) to metallic Bi [S5, S6]. The resulting Bi atoms then grew into two-dimensional bismuth nanosheets anchored and confined within the NCL framework. The final product, denoted as the BiNS/NCL heterostructure, was collected by centrifugation at 8000 rpm for 10 min, washed thoroughly with ethanol and deionized water, and vacuum‑dried at 60 °C overnight.

For comparison, bulk BiNS were synthesized via a synthesized via a modified polyol‑mediated chemical reduction method [S7]. In a typical procedure, 1.0 g of BiCl_3_ (3.17 mmol) was dissolved in 20 mL of ethylene glycol under vigorous stirring at 60 °C to obtain a clear precursor solution. A freshly prepared aqueous NaBH_4_ solution (10 mL, 0.05 mol L^-1^) was introduced dropwise at a controlled rate of 1.0 mL·min^-1^, initiating an immediate reduction accompanied by a color change from transparent to dark brown. The mixture was maintained at 60 °C under continuous stirring for 2 h to ensure complete crystallization and structural maturation. The resulting black precipitate was isolated by centrifugation at 8000 rpm for 10 min, followed by three alternating wash cycles with ethanol and deionized water to remove residual glycol and inorganic impurities. The final product was vacuum-dried at 60 °C for 12 h, yielding bulk BiNS.

***S1.6. Electrochemical measurements***

Electrochemical measurements, including cyclic voltammetry (CV), galvanostatic charge/discharge (GCD), and electrochemical impedance spectroscopy (EIS), were performed at room temperature using a three‑electrode configuration with 1 mol L^-1^ NaCl aqueous electrolyte on a CHI 760E electrochemical workstation (Chenhua, Shanghai, China). A Pt/C electrode and a KCl‑saturated Ag/AgCl electrode served as the counter and reference electrodes, respectively. The working electrode slurry was prepared by ultrasonically dispersing 2 mg of the as‑synthesized sample in 950 µL of isopropanol and 50 µL of Nafion solution (5.0 wt.%) at room temperature for 30 min. The resulting homogeneous slurry was then coated onto carbon paper (2 × 0.5 cm^2^) and vacuum‑dried at 60 °C for 12 h. All electrochemical measurements were conducted in 30 mL of electrolyte at 25 °C.

The specific capacitances (*C*, F g^-1^) were calculated by the following Eq. (1) from the GCD curves [S8, S9]:

$\text{C}\text{=}\frac{\text{I×∆t}}{\text{m×V}}$ [S1]

where *I* is the current (A), ∆*t* is the discharge time (s), *m* is the mass of sample (g), and *V* is the voltage window (V).

The correlation between the measured current (*i*) and the scanning rate (*v*) can be expressed by the following Eqs. (2) and (3) [S10, S11]:

$\text{i}\text{=a}\text{v}^{\text{b}}$ [S2]

$\log\left( \text{i} \right)\text{=blog}\left( \text{v} \right)\text{+log(a)}$ [S3]

where the fitting parameters a and b, obtained from the fitted curves, can indicate whether the process is diffusion-controlled (when b approaches 0.5) or capacitive-controlled (when b is close to 1.0) [S11, S12].

The contribution mechanisms of various charging and discharging processes are calculated by the following Eq. (4):

$\text{i}\left( \text{V} \right)\text{=}\text{k}_{\text{1}}\text{v+}\text{k}_{\text{2}}\text{v}^{\text{1/2}}$ [S4]

where *k*_1_*v* and *k*_2_*v*^1/2^ represent the surface-controlled (i.e., capacitive contribution) and diffusion-controlled (i.e., Faradaic contribution) processes [S10, S11], respectively.

***S1.7. Capacitive deionization measurements***

CDI measurements were performed in batch‑mode with continuous solution recirculation using a cell consisting of a BiNS/NCL Faradaic anode, an activated carbon (AC) cathode, anion‑ and cation‑exchange membranes, a peristaltic pump, a DC power supply, and a reservoir. Each CDI electrode was fabricated by coating a homogeneous slurry onto graphite paper (2.5 × 2.5 cm^2^), followed by vacuum‑drying at 60 °C for 12 h. For the anode, the slurry was composed of the active material (e.g., BiNS/NCL, 16 mg), Super‑P conductive carbon (2 mg), and PVDF binder (2 mg) at a weight ratio of 8: 1: 1, dispersed in 2 mL of NMP. For the cathode, the same procedure was followed using activated carbon (AC, 16 mg) as the active material, with all other components and conditions kept identical.

During CDI testing, the NaCl concentration change in the effluent was monitored in real‑time using a conductivity meter under varied initial concentrations (50, 100, 250, 500, and 1000 mg L^-1^) and applied voltages (0.8, 1.0, 1.2, 1.4, and 1.6 V). The total solution volume was maintained at 32 mL and circulated at a flow rate of 30 mL min^-1^. The Cl^-^ adsorption capacity (SAC_Cl_^-^, mg g^-1^) and Cl^-^ adsorption rate (SAR_Cl_^-^, mg g^-1^ min^-1^) at *t* min were calculated according to Eqs. (5) and (6) [S12, S13]:

$\text{SAC}\text{Cl}\text{-}\text{=}\frac{\text{(}\text{C}_{\text{0}} \text{-}\text{ }\text{C}_{\text{t}}\text{)×V}}{\text{m}}$ [S5]

$\text{SA}\text{R}\text{Cl}\text{-}\text{=}\frac{\text{SAC}}{\text{t}}$ [S6]

where *C_0_* and *C_t_* are the NaCl concentrations at the initial stage and *t* min (mg L^-1^), respectively; *V* is the solution volume (L); and *m* is the total mass of the BiNS/NCL on the working electrodes.

The charge efficiency (*Λ*) was calculated according to Eq. (7) [S14]:

$\text{Λ=}\frac{\text{Γ}\text{ }\text{×}\text{ }\text{F}}{\text{∑}}$ [S7]

where *Γ* (mol g^-1^) is the desalination capacity, *F* is the Faraday constant (96485 C mol^-1^), and *∑* (Charge, C g^-1^) is obtained by integrating current.

The energy consumption (*E*, Wh g^-1^) is obtained according to Eq. (8) [S15]:

$\text{E=}\frac{\text{v×}\int\text{idt}}{\text{3.6×(}\text{C}_{\text{0}} \text{-}\text{ }\text{C)V}}$ [S8]

where *v* is the applied voltage (V), $\int\text{idt}\text{ }$ represents the integrated current over time (C), *V* is the volume of the reaction solution (mL), *C_0_* and *C* are the initial and final concentrations (mg L^-1^) [S15, S16], respectively.

***S1.8. Cl^-^ removal selectivity***

The Cl^-^ removal selectivity of BiNS/NCL was systematically evaluated in a simulated brackish water system containing equimolar concentrations (10 mmol L^-1^ each) of competing anions, including F^-^, Br^-^, NO_3_^-^, and SO_4_^2-^ ions, alongside the target Cl^-^ ions. To quantify the preferential adsorption of Cl^-^ over coexisting anions, the corresponding ion selectivity coefficients (e.g., $\text{S}_{\text{F}^{\text{-}}}^{\text{Cl}^{\text{-}}}$, $\text{S}_{\text{Br}^{\text{-}}}^{\text{Cl}^{\text{-}}}$, $\text{S}_{\text{NO}_{\text{3}}^{\text{-}}}^{\text{Cl}^{\text{-}}}$, and $\text{S}_{\text{NO}_{\text{3}}^{\text{-}}}^{\text{Cl}^{\text{-}}}$) were determined using Eq. (9) [S17, S18], where for instance the selectivity coefficient of Cl^-^ relative to F^-^ is given by:

$\text{S}_{\text{F}^{\text{-}}}^{\text{Cl}^{\text{-}}}\text{=}\frac{\text{C}_{\text{Cl}^{\text{-}}}^{\text{0}}\text{-}\text{C}_{\text{Cl}^{\text{-}}}}{\text{C}_{\text{Cl}^{\text{-}}}^{\text{0}}}\text{/}\frac{\text{C}_{\text{F}^{\text{-}}}^{\text{0}}\text{-}\text{C}_{\text{F}^{\text{-}}}}{\text{C}_{\text{F}^{\text{-}}}^{\text{0}}}$ [S9]

where $\text{C}_{\text{Cl}^{\text{-}}}^{\text{0}}$ and $\text{C}_{\text{Cl}^{\text{-}}}$ (μmol L^-1^) are the initial and final Cl^-^ concentrations, while $\text{C}_{\text{F}^{\text{-}}}^{\text{0}}$ and $\text{C}_{\text{F}^{\text{-}}}$ (μmol L^-1^) are the initial and final F^-^ concentrations [S19, S20], respectively.

***S1.9.*** *In-situ EIS measurements and DRT analysis*

The distribution of relaxation times (DRT) analysis was subsequently employed to deconvolute the complex electrochemical processes of BiNS/NCL by transforming the frequency-dependent impedance and admittance data into the time domain. DRT impedance, Z_DRT_ (*f*), which represents the characteristic relaxation time distribution at frequency *f*, can be mathematically described by Eq. (10) [S21, S22]:

$\text{Z}_{\text{DRT}}\left( \text{f} \right)\text{=}\text{i}\text{2π}\text{f}\text{L}_{\text{0}}\text{+}\text{R}_{\text{∞}}\text{+}\int_{\text{-∞}}^{\text{+∞}} \frac{\text{γ}\left( \log\text{τ} \right)}{\text{1+}\text{i}\text{2π}\text{f}\text{τ}}\text{d}\log\text{τ}$ [S10]

where *L*_0_, *R*_∞_, *τ*, and *γ*(log*τ*) are an inductance, an ohmic resistance, a timescale, and the DRT, respectively. In turn, the total polarization resistance, *R*_pol_, was computed using the following Eq. (11) [S21, S22]:

$\text{R}_{\text{pol}}\text{=}\int_{\text{-∞}}^{\text{+∞}} \text{γ}\left( \log\text{τ} \right)\text{d}\log\text{τ}$ [S11]

**S2. Computational Methods**

All first-principles calculations were performed using the plane-wave pseudopotential method as implemented in the Cambridge Sequential Total Energy Package (CASTEP) within the Materials Studio software suite [S23, S24]. The exchange-correlation interactions were described within the generalized gradient approximation (GGA) employing the Perdew-Burke-Ernzerhof (PBE) functional. Core-valence interactions were treated using optimized ultrasoft pseudopotentials. A kinetic energy cutoff of 517 eV was applied for the plane-wave basis set to ensure converged total energies and electronic structures. Reciprocal space integration was carried out using a Γ-centered k-point mesh with a spacing of 0.015 Å^-1^, generated via the Monkhorst-Pack scheme, which provides sufficient sampling of the Brillouin zone for density of states (DOS) analysis.

Structural optimizations were performed for all models, including the bulk BiNS, NCL, and BiNS/NCL. To eliminate artificial interactions between periodic images, a vacuum layer of at least 18 Å was inserted along the non-periodic direction perpendicular to the interface plane. The geometry relaxation proceeded until the following convergence criteria were satisfied: (1) total energy change per atom < 1.0 × 10^-5^ eV, (2) maximum residual force on any atom < 0.02 eV Å^-1^, (3) maximum atomic displacement < 0.001 Å, and (4) maximum stress component < 0.05 GPa. Subsequently, self-consistent field calculations with an energy convergence threshold of 1.0 × 10^-6^ eV were performed to obtain accurate electronic densities and DOS profiles, ensuring that the Hellmann-Feynman forces on all atoms remained below 0.03 eV Å^-1^.

**S3. Work Function Calculations**

The electronic work function (*Φ*) was determined using DFT as implemented in the CASTEP code [S25, S26]. Ultrasoft pseudopotentials were employed to describe core electrons, while the exchange-correlation interactions were treated using the PBE functional under the GGA. To model the surface and eliminate artificial interactions between periodic images along the non-periodic direction, a vacuum layer of at least 18 Å was introduced normal to the surface plane.

After achieving self-consistent electronic ground states, the macroscopic average of the electrostatic potential was computed along the surface normal direction. The vacuum energy level (*E_vac_*) was identified as the plateau region of the planar-averaged electrostatic potential in the vacuum region, sufficiently far from the slab. The work function was then calculated as the energy difference between the vacuum level and the Fermi energy (*E_F_*) according to the following Eq. [12] [S25, S26]:

*Φ* = *E_vac_ - E_F_* [S12]

where *E_F_* corresponds to the Fermi level of the slab model and *E_vac_* is the converged vacuum electrostatic potential. All calculations were carried out with a kinetic energy cutoff of 400 eV and a *Γ*-centered *k-*point grid with a spacing of 0.03 Å^-1^. Systematic tests on slab thickness and vacuum layer size confirmed that the calculated work function values converged within 0.05 eV.


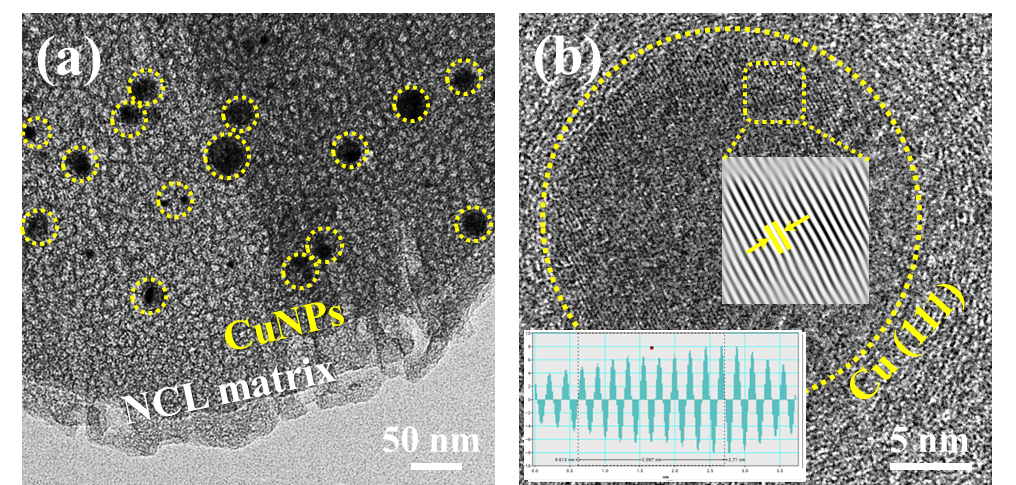


**Fig. S1**. (a) TEM and (b) HRTEM images of CuNP/NCL.

**Fig. S2**. Optical micrographs of the water contact angles on the surface of (a) CuNP/NCL, (b) BiNS, and (c) BiNS/NCL.

**Table S1**.

Modeling and calculation of water molecule adsorption on CuNP/NCL, BiNS, and BiNS/NCL.

| **Models** | 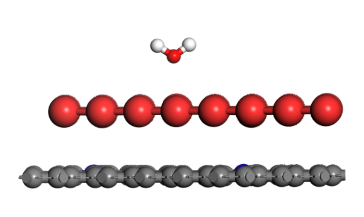  **CuNP/NCL** | 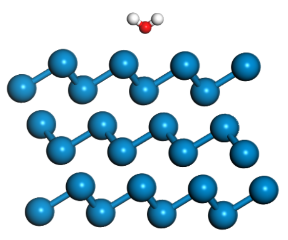  **BiNS** | 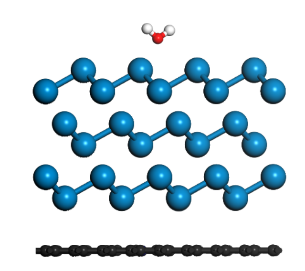  **BiNS/NCL** |
| --- | --- | --- | --- |
| Total energy after water molecule absorption (eV) | -128220.29 | -38396.43 | -58603.55 |
| Total energy before water molecule absorption (eV) | -127748.42 | -37924.70 | -58131.47 |
| Total energy of H_2_O (eV) | -471.60 | -471.60 | -471.60 |
| Adsorption energy (eV) | -0.27 | -0.13 | -0.48 |

**Fig. S3**. High-resolution XPS spectra of (a) Cu 2p and (b) N 1s for CuNP/NCL and BiNS/NCL.

**Fig. S4**. The electrostatic potentials of (a) bulk BiNS and (b) NCL.

**Fig. S5**. (a, b) SEM images and (c) XRD patterns of bulk BiNS.

**Fig. S6**. CV curves of (a) CuNP/NCL and (b) bulk BiNS at the scan rates ranging from 1 to 10 mV s^-1^.

**Fig. S7**. GCD curves of (a) CuNP/NCL and (b) bulk BiNS at the current densities from 1 to 10 A g^-1^.

**Fig. S8**. (a) Normalized proportions of surface- and diffusion-controlled contributions for bulk BiNS. (b) Decoupling of surface- and diffusion-controlled contributions for bulk BiNS at a scan rate of 1 mV s^-1^.

**Fig. S9**. Time‑dependent SAC_Cl-_ profiles versus dechlorination time for (a) CuNP/NCL and (b) bulk BiNS with initial Cl^-^ concentration of 500 mg L^-1^ under different voltages from 0.8 to 1.6 V.

**Fig. S10**. The CDI Ragone plots of (a) BiNS/NCL, (b) CuNP/NCL and (c) bulk BiNS with initial Cl^-^ concentration of 500 mg L^-1^ under different voltages from 0.8 to 1.6 V.

**Fig. S11**. Time‑dependent SAC variations and corresponding CDI Ragone plots. (a-c) Time‑resolved SAC profiles and (d-f) corresponding CDI Ragone plots for (a, d) CuNP/NCL, (b, e) bulk BiNS, and (c, f) BiNS/NCL electrodes measured under an applied voltage of 1.2 V at initial NaCl concentrations ranging from 50 to 1000 mg L^-1^.

**Fig. S12.** Langmuir isotherm and experimental SAC_Cl_^-^ values of BiNS/NCL in the Cl^-^ concentrations ranging from 50 to 1000 mg L^-1^ under an applied voltage of 1.2 V.

**Table S2**.

Coefficients of Langmuir fitting.

| **Isotherm** | **Model equation** | **Parameter** | **Value** |
| --- | --- | --- | --- |
| Langmuir | $\text{q=}\frac{\text{q}_{\text{m}}\text{K}_{\text{L}}\text{C}}{\text{1}\text{ }\text{+}\text{ }\text{K}_{\text{L}}\text{C}}$ | *q_m_* | 97.8 |
|  |  | *K*_L_ | 0.0132 |
|  |  | *r*^2^ | 0.997 |

**Fig. S13**. Post-cycling (a) TEM and (b) HRTEM characterizations of BiNS/NCL after 100 dechlorination/regeneration cycles.

**Fig. S14**. XRD patterns of BiNS/NCL before and after 100 dechlorination/regeneration cycles.

**Fig. S15**. (a) High-resolution Bi 4f XPS spectra of BiNS/NCL before and after 100 dechlorination/regeneration cycles and (b) corresponding integrated peak area ratios of Bi^0^ 4f_5/2_ and Bi^0^ 4f_7/2_ before and after cycling. Note: The integrated peak area ratios were calculated by dividing the individual peak area of Bi^0^ 4f_5/2_ or Bi^0^ 4f_7/2_ by the sum of both peak areas.

**Table S3**. Comparison of the Cl^-^ capture capacity for BiNS/NCL with previously reported Faradaic dechlorination electrodes.

| **Electrode materials^a^** | **NaCl concentrations**  **(mg L^-1^)** | **Voltage (V)**/**Specific current (mA g^-1^)** | **SAC_Cl-_**  **(mg g^-1^)** | **Refs** |
| --- | --- | --- | --- | --- |
| Ag@DMCS | 1000 | 1.5 | 68.74 | [S27] |
| Ag@CNP | 824 | 1.2 | 77.73 | [S28] |
| AGC | 1000 | 1.2 | 62.4 | [S29] |
| Ag@PANI | 500 | 1.4 | 25.1 | [S30] |
| Ag/AC | 584.4 | 1.2 | 23.3 | [S31] |
| Ag@rGO | 2000 | 1.4 | 82.2 | [S32] |
| Ag/ZCs | 500 | 1.2 | 29.18 | [S33] |
| Bi-Fc-MOF | 500 | 1.4 | 74.07 | [S34] |
| Bi | 500 | 1.2 | 53.82 | [S17] |
| CS@BiOCl | 3000 | (800 mA g^-1^) | 52.67 | [S35] |
| Bi@MXene | 584.4 | (20 mA g^-1^) | 61.0 | [S36] |
| BiNS@MXene | 1160 | 1.2 | 88.2 | [S4] |
| Bi NCs@CNF | 3000 | (1300 mA g^-1^) | 102.26 | [S37] |
| BiOCl | 500 | 1.2 | 74.75 | [S38] |
| Bi@C | 1000 | 1.2 | 75.3 | [S39] |
| Bi@CB | 1000 | 1.2 | 38.9 | [S40] |
| TpPy(Me)Cl@GO | 600 | 1.4 | 62.0 | [S41] |
| C/CoNi-LDH | 1000 | 1.4 | 60.88 | [S42] |
| NiFe-LDO/C | 250 | 1.2 | 43.68 | [S43] |
| ZnCo-Cl LDH | 500 | 1.2 | 56.1 | [S44] |
| Cl-FeOOH | 500 | 1.2 | 42.62 | [S45] |
| Cu@CHs | 500 | 1.4 | 44.42 | [S46] |
| TiO_2_/CNTs | 200 | 1.2 | 6.5 | [S47] |
| NPC | 500 | 1.2 | 21.4 | [S48] |
| Ag@GNS | 1000 | 1.4 | 88.2 | [S49] |
| TAPT-BDB-COF | 500 | 1.5 | 87.2 | [S50] |
| Fe-CNF | 500 | 1.2 | 82.5 | [S51] |
| HCSs@FeOOH | 3000 | (225 mA g^-1^) | 69.48 | [S52] |
| LDH-SDS | 500 | 1.2 | 61.4 | [S53] |
| N-a_4_pC | 500 | 1.2 | 38.1 | [S54] |
| NPC/NiMn-LDH/MXene | 500 | 1.2 | 43.5 | [S55] |
| PPy-NiCo-LDH@MXene | 500 | 1.2 | 31.5 | [S56] |
| TAPArGO-75 | 500 | (100 mA g^-1^) | 55.0 | [S57] |
| AgCuG | 600 | 1.2 | 16.8 | [S58] |
| BiOCl@G | 500 | 1.4 | 87 | [S59] |
| CNF@Mn_2_O_3_ | 3000 | 1.2 | 27.43 | [S60] |
| CNT@NaMnO_2_ | 500 | 1.2 | 32.7 | [S61] |
| MgAl-O*_x_*/G | 500 | 1.0 | 13.6 | [S62] |
| MoS_2_@MXene | 500 | 1.2 | 35.6 | [S63] |
| MXene@COF | 1000 | 1.2 | 37.2 | [S64] |
| NiCoFe-PBA/CNT | 500 | 1.4 | 70.4 | [S65] |
| KCu[Fe(CN)_6_] | 500 | 1.2 | 23.2 | [S66] |
| MXene/BC@PPy | 1168.8 | 1.2 | 17.56 | [S67] |
| CNFAs@FeOOH | 3000 | 1.2 | 66.7 | [S68] |
| CNTs@FeOOH | 3000 | (900 mA g^-1^) | 50.36 | [S69] |
| BiNS/NCL | 1000 | 1.2 | 88.3 | This study |
|  | 500 | 1.6 | 108.7 |  |

^a^ **Ag@DMCS**: Ag-decorated monolithic carbon sponge; **Ag@CNP**: Ag@nitrogen-phosphorus co-doped carbon composites; **AGC**: Ag@graphene/carbon nanotubes; **Ag@PANI**: Ag-coated polyaniline-modified activated carbon; **Ag/AC**: Ag-coated activated carbon; **Ag@rGO**: Ag/reduced graphene oxide; **Ag/ZCs**: Ag-doped hollow ZIFs-derived nanoporous carbon; **Bi-Fc-MOF**: Conductive porous Bi-based metal-organic framework nanoflowers with dual redox active sites; **Bi**: Pristine Bi electrode; **CS@BiOCl**: BiOCl nanostructure coated carbon sponge; **Bi@MXene**: Bi@ Ti_3_C_2_T*_x_* MXene free-standing film electrode; **BiNS@MXene**: Hierarchical bismuthine nanosheets@MXene; **Bi NCs@CNF**: Ultrasmall Bi nanoclusters impregnated carbon nanofibers; **BiOCl**: BiOCl through crystal plane engineering; **Bi@C:** Hierarchical bismuth-embedded carbon; **Bi@CB**: Addition of carbon black to a Bi electrode; **TpPy(Me)Cl@GO**: Cationic *β*-ketoenamine 2D 1,3,5-triformylphloroglucinol-2,5-diaminopyridine-covalent organic framework; **C/CoNi-LDH**: Hollow nanocage structure of CoNi-layered double hydroxide/carbon composites; **NiFe-LDO/C**: NiFe layered double oxides/carbon; **ZnCo-Cl LDH**: ZnCo-layered double hydroxide nanoclusters with interlayer-bonded chloride ions; **Cl-FeOOH**: FeOOH hybrid heterostructures with Cl dopants; **Cu@CHs**: the Cu/ Cu_2_O@PVA/CNTs conductive hydrogels; **TiO_2_/CNTs**: TiO_2_/carbon nanotubes; **NPC**: Polymerization of m-phenylenediamine/phytic acid-derived N/P co-doped carbon; **Ag@GNS:** Ag-graphene nanoscroll; **TAPT-BDB-COF**: a cationic viologen-based 2D 4,4*'*,4*''*-(1,3,5-triazine-2,4,6-triyl)trianiline-1,1*'*-bis(2,4-dinitrophenyl)-4,4*'*-bipyridinium dichloride-covalent organic framework; **Fe-CNFs**: Iron nanoparticle embedded carbon nanofibers; **HCSs@FeOOH**: 3D sea urchin-like hollow carbon spheres@FeOOH nanorod; **LDH-SDS**: Sodium dodecyl sulfate (SDS) intercalated layered double hydroxides; **N-a_4_pC**: N-doped activated porous carbon; **NPC/NiMn-LDH/MXene**: N-doped porous carbon spheres/NiMn-layered double hydroxide/MXene; **PPy-NiCo-LDH@MXene**: Polypyrrole-NiCo-layered double hydroxides@MXene; **TAPArGO**: *N,N,N′,N′*-tetra(p-aminophenyl)-p-phenylenediamine (TAPD) and 4,4′,4″,4^‴^-(1,4-phenylene-bis (azanetriyl))tetrabenzaldehyde-COF/conductive graphene; **AgCuG**: Bimetallic silver-copper decorated graphene nanocomposite; **BiOCl@G**: Ultrasmall BiOCl nanoclusters on graphene sheets; **CNF@Mn_2_O_3_**: Mn_2_O_3_ nanoflower decorated carbon nanofibers; **CNT/NaMnO_2_**: Carbon nanotube/MnO_2_ nanosheets with Na^+^ ions; **MgAlO*_x_*/G**: Calcined MgAl-layered double hydroxide/graphene hybrids; **MoS_2_@MXene**: Few-layered MXene/MoS_2_ nanosheets; **MXene@COF**: Ti_3_C_2_T*_x_* MXene/*β*-ketoenamine-linked covalent organic framework; **NiFeCo-PBA/CNT**: Ni, Co, Fe,-ternary-metal Prussian blue analogue/carbon nanotube; **KCu[Fe(CN)_6_]**: Cu/potassium ferricyanide metal organic framework; **MXene/BC@PPy:** Ti_3_C_2_T*_x_* MXene/Bacterial cellulose nanofibers@polypyrrole; **CNFAs@FeOOH**; Carbon nanofiber aerogels@FeOOH nanospindle; **CNTs@FeOOH**; Chloride-insertion FeOOH/carbon nanotubes.

**Table S4**. Modeling and calculation of Cl^-^ adsorption on BiNS/NCL and BiNS.

| **Models** | 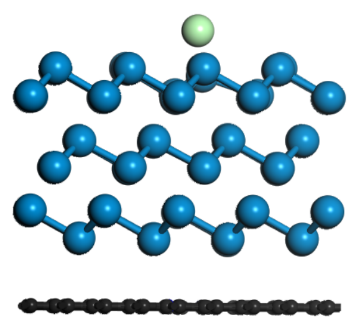  BiNS/NCL | 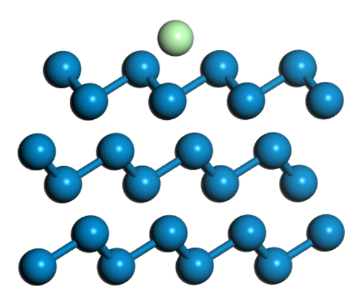  BiNS |
| --- | --- | --- |
| Total energy of after Cl absorption (eV) | -58560.94 | -38351.56 |
| Total energy of before Cl absorption (eV) | -58131.47 | -37922.70 |
| Total energy of Cl atom (eV) | -425.93 | -425.93 |
| Adsorption energy (eV) | -3.54 | -2.93 |

**Fig. S16**. Schematic diagram of the chloride diffusion path on the surface of (a) bulk BiNS and (b) BiNS/NCL at different directions, respectively.

**References**

[S1] K. Yang, S. Hu, Y. Ban, Y. Zhou, N. Cao, M. Zhao, Y. Xiao, W. Li and W. Yang, ZIF-L membrane with a membrane-interlocked-support composite architecture for H_2_/CO_2_ separation, *Sci. Bull.* 2021, 66, 1869.

[S2] S. Xie, X. Tan, N. Chanut, Z. Zhou, W. Monens, X. Zhang, H. Pan, H. Pan, X. Zhang, I. F. J. Vankelecom and J. Fransaer, Tuning surface concentrations of building units to enable the cathodic deposition of metastable ZIF-L films for gas separations, *Angew. Chem. Int. Ed.* 2025, 64, e202511638.

[S3] J. Li, W. Xia, T. Wang, L. Zheng, Y. Lai, J. Pan, C. Jiang, L. Song, M. Wang, H. Zhang, N. Chen, G. Chen and J. He, A facile route for constructing effective Cu-N*_x_* active sites for oxygen reduction reaction, *Chem. Eur. J.* 2020, 26, 4070.

[S4] S. Gong, H. Liu, F. Zhao, Y. Zhang, H. Xu, M. Li, J. Qi, H. Wang, C. Li, W. Peng, X. Fan and J. Liu, Vertically aligned bismuthene nanosheets on MXene for high-performance capacitive deionization, *ACS Nano* 2023, 17, 4843.

[S5] Y. Wang, X. Xu, Y. Wu, F. Li, W. Fan, Y. Wu, S. Ji, J. Zhao, J. Liu and Y. Huo, Facile galvanic replacement construction of Bi@C nanosheets array as binder-free anodes for superior sodium-ion batteries, *Adv. Energy Mater.* 2024, 14, 2401833.

[S6] S. Yang, X. Gu, X. Feng, R. Wang, X.-Y. Lou, W. Wei and H. Chen, Engineering the reversible redox electrochemistry on cuprous oxide for efficient chloride ion uptake, *Nat. Commun.* 2025, 16, 2282.

[S7] K. O. Adeniyi, G. Manavalan, A. Zainelabdin, J.-P. Mikkola and S. Tesfalidet, Solution-processable bismuthene nanosheets for ultrasensitive sensing of heavy metal ions via anodic stripping voltammetry, *ACS Appl. Nano Mater.* 2024, 7, 20217.

[S8] P. Li, X. Chong, Y. Guo, G. Wu, S. Wang, R. Liu, J. Ma, Y. Wang, W. Chen, Y. Cai, Y. Wang, Q. Yuan and J. Zhao, Heterostructure-engineered conductive MOFs coupled with MoSe_2_@MXene framework for efficient and selective lithium extraction from salt lake brine via capacitive deionization, *Adv. Sci.* 2026, 13, e21830.

[S9] Y. Qiao, Y. Li, C. Wang, Q. Pan, B. Chen and Y. Wang, Layered double oxide as a Cl^-^ selective anode for high-performance electrochemical lithium extraction, *ACS Sustain. Chem. Eng.* 2024, 12, 11692.

[S10] Q. Lu, H. Duan, K. Zhang, H. Wang, D. Han, Z. Wang, Y. Xu, Q. Yi, Y. Shen, T. Yan, M. Xie and D. Zhang, Regulation of intra-nanopore microenvironment in oxygen-rich covalent organic frameworks for enhanced capacitive deionization, *Adv. Funct. Mater.* 2025, 35, 2507253.

[S11] C. He, J. Zhang, D. Mantzavinos, A. Katsaounis, D.-H. Si, Z. Yan, H.-Y. Zhang and Z.-W. Jiang, Elaborate designed three-dimensional hierarchical conductive MOF/LDH/CF nanoarchitectures for superior capacitive deionization, *Angew. Chem. Int. Ed.* 2025, 64, e202420295.

[S12] C. Tang, H. Chen, Q. Li, C. Li, Y. Li, A. Alowasheeir, Z. M. El-Bahy, G. Wang, C. Zhang, Y. Yamauchi and X. Xu, Directional ion transport through nanoarchitected 1D mesochannels: 2D polymer interfacial engineering for high-efficiency capacitive deionization, *Adv. Sci.* 2025, 12, e04527.

[S13] J. Peng, X. Zhao, Y. Huang, C. Zhao, J. Yang and M. Shi, Electron-delocalized benzimidazole-linked organic electrode with enhanced redox availability for high-performance ammonium capture via capacitive deionization, *Adv. Funct. Mater.* 2025, 35, 2506369.

[S14] S. Luo, L. Yang, D. Guo, K. Tang, Z. Duan, S. Zhu, Y. Chen, X. Liu, Y. Dong, A. Kanwal, S. Liu, L. Yao, B. Liu, G. Shao, K. Zhou and H. Wu, Silver-decorated vacancy-rich mesopore MXene membrane for efficient chloride removal via CDI, *Chem. Eng. J.* 2025, 525, 169883.

[S15] W. Kong, Y. Wang, K. Tan, X. Lu, M. Fang, Z. Che, M. Zhang and Y. Feng, Air-plasma synthesis of highly-dispersed noble-metal nanocrystals encapsulated in graphene nanoscroll: with Ag-graphene as an example for capacitive deionization, *Chem. Eng. J.* 2026, 527, 171487.

[S16] F. Ma, L. Li, X. Chen, X. He, Q. Li, J. Sun, R. Jiang, Z. Lei and Z.-H. Liu, A bipartite synergistic strategy for all-weather sodium-ion fiber supercapacitor with excellent energy density and temperature adaptability, *Adv. Funct. Mater.* 2025, 35, 2506540.

[S17] J. Chang, Y. Li, F. Duan, C. Su, Y. Li and H. Cao, Selective removal of chloride ions by bismuth electrode in capacitive deionization, *Sep. Purif. Technol.* 2020, 240, 116600.

[S18] Y. Xu, H. Zhou, G. Wang, Y. Zhang, H. Zhang and H. Zhao, Selective pseudocapacitive deionization of calcium ions in copper hexacyanoferrate, *ACS Appl. Mater. Interfaces* 2020, 12, 41437.

[S19] F. Pang, B. Liu, J. Wu, Q. Yang, Z. Xiao, N. Shang, X. Zhao, Y. Yamauchi and S. Zhang, Dual-phased molybdenum carbides confined in MOF-derived carbon nanoframes enhance capacitive desalination, *Adv. Funct. Mater.* 2026, 36, e17130.

[S20] W. Song, B. Li, Y. Qu, W. Jiang, M. Pei, N. Hu, S. Zhuo, C. Su, X. Jin, R. Mao, D. Liu, X. Jian and F. Hu, Dynamic migration-pulling polymer electrolyte design strategy for low-temperature lithium-sulfur batteries, *Angew. Chem. Int. Ed.* 2025, 64, e202505095.

[S21] J. Chen, E. Quattrocchi, F. Ciucci, Y. Chen, Charging processes in lithium-oxygen batteries unraveled through the lens of the distribution of relaxation times, *Chem* 2023, 9, 2267-2281.

[S22] X. Cai, C. Zhang, H. Ruan, Z. Chen, L. Zhang, D.U. Sauer, W. Li, Cross-scale decoupling kinetic processes in lithium-ion batteries using the multi-dimensional distribution of relaxation time. *Adv. Sci.* 2024, 11, 2406934.

[S23] H. Arif, M. B. Tahir, B. S. Almutairi, I. Khalid, M. Sagir, H. Elhosiny Ali, H. Alrobei and M. Alzaid, CASTEP study for mapping phase stability, and optical parameters of halide perovskite CsSiBr_3_ for photovoltaic and solar cell applications, *Inorg. Chem. Commun.* 2023, 150, 110474.

[S24] M. D. Segall, J. D. L. Philip, M. J. Probert, C. J. Pickard, P. J. Hasnip, S. J. Clark, M. C. Payne, First-principles simulation: ideas, illustrations and the CASTEP code, *J. Phys. Condens. Matter,* 2002, 14, 2717.

[S25] Z. Chen, T. Ma, W. Wei, W.-Y. Wong, C. Zhao, B.-J. Ni, Work function-guided electrocatalyst design. *Adv. Mater.* 2024, 36, 2401568.

[S26] A. Kahn, Fermi level, work function and vacuum level, *Mater. Horiz.* 2016, 3, 7-10.

[S27] L. Yan, B. Wu, Y. Zhao, J. Annor Asare, H. Gang, D. Wei, Y. Cao, P. Chen, H. Wang and L. Huang, Ag-decorated monolithic carbon sponge with porous directional frameworks for chloride removal in flow-through capacitive deionization, *Sep. Purif. Technol.* 2023, 322, 124210.

[S28] Y. Cao, L. Yan, B. Wu, D. Wei, B. Ouyang, P. Chen, T. Zhang, J. A. Asare, Y. Jiang, Y. He and H. Wang, Enhancement of interfacial interaction between carbon and silver nanoparticles through co-doping with nitrogen and phosphorus, *Desalination* 2024, 581, 117603.

[S29] V. M. Rangaraj, J.-I. Yoo, J.-K. Song and V. Mittal, MOF-derived 3D MnO_2_@graphene/CNT and Ag@graphene/CNT hybrid electrode materials for dual-ion selective pseudocapacitive deionization, *Desalination* 2023, 550, 116369.

[S30] B. Wei and Z. Liu, Pseudo-capacitive behaviors induced dual-ion selective deionization system based on MoS_2_/PPy//Ag@PANI/AC, *Sep. Purif. Technol.* 2025, 362, 131906.

[S31] H. Yoon, J. Lee, T. Min, G. Lee and M. Oh, High performance hybrid capacitive deionization with a Ag-coated activated carbon electrode, *Environ. Sci.: Water Res. Technol.* 2021, 7, 1315-1321.

[S32] Z. Yue, Y. Ma, J. Zhang and H. Li, Pseudo-capacitive behavior induced dual-ion hybrid deionization system based on Ag@rGO ‖ Na_1.1_V_3_O_7.9_@rGO, *J. Mater. Chem. A* 2019, 7, 16892-16901.

[S33] H. Zhang, W. Zhang, J. Shen, Y. Li, X. Yan, J. Qi, X. Sun, J. Shen, W. Han, L. Wang and J. Li, Ag-doped hollow ZIFs-derived nanoporous carbon for efficient hybrid capacitive deionization, *Desalination* 2020, 473, 114173.

[S34] D. Wei, H. Dong, B. Ouyang, P. Chen, T. Zhang, Y. He, L. Huang and H. Wang, Conductive bismuth-based metal-organic frameworks with dual redox sites for efficient capacitive deionization, *Chem. Eng. J.* 2025, 513, 162947.

[S35] K. Wang, X. Du, Z. Liu, B. Geng, W. Shi, Y. Liu, X. Dou, H. Zhu, L. Pan and X. Yuan, Bismuth oxychloride nanostructure coated carbon sponge as flow-through electrode for highly efficient rocking-chair capacitive deionization, *J. Colloid Interf. Sci.* 2022, 608, 2752.

[S36] Z. Tan, M. Xu, X. Ji and Z. He, Construction of anode Bi@Ti_3_C_2_T*_x_* MXene free-standing electrode for high-efficiency hybrid capacitive deionization, *J. Environ. Chem. Eng.* 2025, 13, 119613.

[S37] L. Wang, Z. Liu, Z. Wang, Q. Ma, Z. Guo, G. Shen, K. Wang, X. Xu, Y. Liu and X. Yuan, Up-shifting the desalination rate limit of capacitive deionization via integrating chloride-capturing Bi nanocluster with flow-through cell architecture, *Chem. Eng. J.* 2023, 460, 141726.

[S38] X. Wang, J. Zhou, S. Weng, X. Lu and Y. Xia, Crystal plane engineering of BiOCl for enhanced chloride-ion storage and saline water deionization performances, *Sep. Purif. Technol.* 2025, 369, 133170.

[S39] C. Li, Y. Zhang, S. Gong, Y. Zhang, X. Yan, H. Xu, Z. Cui, J. Qi, H. Wang, X. Fan, W. Peng and J. Liu, Strong interface coupling boosting hierarchical bismuth embedded carbon hybrid for high-performance capacitive deionization, *J. Colloid Interface Sci.* 2023, 648, 357-364.

[S40] J. Chang, F. Duan, C. Su, Y. Li and H. Cao, Removal of chloride ions using a bismuth electrode in capacitive deionization (CDI), *Environ. Sci.: Water Res. Technol.* 2020, 6, 373-382.

[S41] N. M. Aldaqqa, S. Kumar, J. I. Martínez, N. Elmerhi, E. Alhseinat and D. Shetty, Surface engineered 2D-*β*-ketoenamine covalent organic framework for superior dechlorination via hybrid capacitive deionization, *Angew. Chem. Int. Ed.* 2025, 64, e202510345.

[S42] D. Wei, Y. Cao, L. Yan, H. Gang, B. Wu, B. Ouyang, P. Chen, Y. Jiang and H. Wang, Enhanced pseudo-capacitance process in nanoarchitectural layered double hydroxide nanoarrays hollow nanocages for improved capacitive deionization performance, *ACS Appl. Mater. Interfaces* 2023, 15, 24427-24436.

[S43] J. Hu, W. Xi, Y. Zhang, R. Wang, H. Wang, Y. Gong, B. He and J. Jin, Rational design of LDH-derived NiFe layered double oxides as capacitive deionization anode for efficient chlorine ion storage with a “memory effect”, *Appl. Surf. Sci.* 2025, 687, 162289.

[S44] Z. Zhang and H. Li, Promoting the uptake of chloride ions by ZnCo-Cl layered double hydroxide electrodes for enhanced capacitive deionization, *Environ. Sci.: Nano* 2021, 8, 1886-1895.

[S45] J. Zhao, B. Wu, X. Huang, Y. Sun, Z. Zhao, M. Ye and X. Wen, Efficient and durable sodium, chloride-doped iron oxide-hydroxide nanohybrid-promoted capacitive deionization of saline water via synergetic pseudocapacitive process, *Adv. Sci.* 2022, 9, 2201678.

[S46] F. Yu, T. Chen and J. Ma, Cu/Cu_2_O@polyvinyl alcohol/carbon nanotube conductive hydrogel for chloride ion removal in capacitive deionization, *J. Colloid Interface Sci.* 2026, 701, 138663.

[S47] S. Ma, C. Liu, Y. Xu, Y. Tan, D. Yang, F. Wang and L. Ma, TiO_2_ and carbon nanotubes composites modify capacitive deionization anodes to improve the dechlorination efficiency in desulfurization wastewater, *Water Sci. Technol.* 2021, 84, 1228-1244.

[S48] H. Wang, T. Yuan, L. Huang, Y. He, B. Wu, L. Hou, Q. Liao and W. Yang, Enhanced chloride removal of phosphorus doping in carbon material for capacitive deionization: Experimental measurement and theoretical calculation, *Sci. Total Environ.* 2020, 720, 137637.

[S49] W. Kong, Y. Wang, K. Tan, X. Lu, M. Fang, Z. Che, M. Zhang and Y. Feng, Air-plasma synthesis of highly-dispersed noble-metal nanocrystals encapsulated in graphene nanoscroll: with Ag-graphene as an example for capacitive deionization, *Chem. Eng. J.* 2026, 527, 171487.

[S50] J. Zhang, Z. Chen, H. Zhang, Y. Cui, F. Zheng, Y. Duan, L. Guo, D. Jiang, J. Feng, Y. Lin, Y. Yang, D. Feng, P. Tang and Z. Liang, Viologen-based cationic covalent organic frameworks for efficient dechlorination via capacitive deionization, *Adv. Funct. Mater.* 2026, e30236.

[S51] D. Chen, L. Yang, Z. Zhang, Y.-n. Wang, D. Ouyang, H. Zhu and J. Yin, Iron nanoparticle embedded carbon nanofibers as flexible electrodes for selective chloride ions capture in capacitive deionization, *Desalination* 2024, 573, 117175.

[S52] G. Shen, Z. Guo, L. Zhang, Q. Ma, C. Xiao, C. Qin, H. L. H. Chong, P. Y. Moh, Y. Liu and X. Yuan, Rational design of sea urchin-like FeOOH anchored hollow carbon spheres as chloride-insertion electrodes for efficient faradic capacitive deionization, *Sep. Purif. Technol.* 2024, 335, 126034.

[S53] H. Yang, R. Zhang, M. Hou, D. Li, J. Cao, X. Xu, W. Zhou, G. Wen, X. Huang and D. Wang, Organic anion-intercalation boosting intrinsic Cl- capture capability of layered double hydroxide anode for enhanced capacitive deionization, *Green Energy Environ.* 2025, DOI: 10.1016/j.gee.2025.12.001.

[S54] H. Yang, R. Zhang, Z. Liu, X. Xu, Y. Teng, M. Hou, G. Zhang, Y. Hou, G. Wen and D. Wang, Unraveling the chloride ion capture capability of nitrogen-doped porous carbon for capacitive deionization and desalination battery, *Chem. Eng. J.* 2024, 501, 157769.

[S55] Y. Cai, G. Zhao, Q. Yuan and J. Zhao, Elaborate designed sandwich structural faradic material NPC/NiMn-LDH/MXene for enriched ion accessible transfer pathways in capacitive deionization, *Chem. Eng. J.* 2024, 484, 149491.

[S56] Y. Cai, Y. Wang, R. Fang and J. Wang, Flexible structural engineering of PPy-NiCo-LDH@Mxene for improved capacitive deionization and efficient hard water softening process, *Sep. Purif. Technol.* 2022, 280, 119828.

[S57] L. Xu, J. Wang, Y. Li, Y. Liu, X. Xu, Z. Chen, X. Liu and L. Pan, Heterointerfacial charge modulation of p-type covalent organic frameworks on graphene achieving high-performance Cl^-^ ion storage with ultralong cycling life, *Angew. Chem. Int. Ed.* 2025, 64, e202508092.

[S58] M. R. Vengatesan, I. F. Fahmi Darawsheh, B. Govindan, E. Alhseinat and F. Banat, Ag-Cu bimetallic nanoparticle decorated graphene nanocomposite as an effective anode material for hybrid capacitive deionization (HCDI) system, *Electrochim. Acta* 2019, 297, 1052.

[S59] W. Kong, X. Ge, D. Kong, C. Liu, J. Sun, X. Zhu, M. Zhang and Y. Feng, Highly dispersed ultrasmall BiOCl nanoclusters on graphene sheets as high-performance anion-capture electrode for hybrid capacitive deionization, *Desalination* 2024, 573, 117222.

[S60] Y. Liu, X. Gao, L. Zhang, X. Shen, X. Du, X. Dou and X. Yuan, Mn_2_O_3_ nanoflower decorated electrospun carbon nanofibers for efficient hybrid capacitive deionization, *Desalination* 2020, 494, 114665.

[S61] S. Wang, G. Wang, X. Che, S. Wang, C. Li, D. Li, Y. Zhang, Q. Dong and J. Qiu, Enhancing the capacitive deionization performance of NaMnO_2_ by interface engineering and redox-reaction, *Environ. Sci.: Nano* 2019, 6, 2379.

[S62] Q. Ren, G. Wang, T. Wu, X. He, J. Wang, J. Yang, C. Yu and J. Qiu, Calcined MgAl-layered double hydroxide/graphene hybrids for capacitive deionization, *Ind. Eng. Chem. Res.* 2018, 57, 6417.

[S63] Y. Cai, Y. Wang, L. Zhang, R. Fang and J. Wang, 3D Heterostructure constructed by few-layered MXenes with a MoS_2_ layer as the shielding shell for excellent hybrid capacitive deionization and enhanced structural stability, *ACS Appl. Mater. Interfaces* 2022, 14, 2833.

[S64] S. Zhang, X. Xu, X. Liu, Q. Yang, N. Shang, X. Zhao, X. Zang, C. Wang, Z. Wang, J. G. Shapter and Y. Yamauchi, Heterointerface optimization in a covalent organic framework-on-MXene for high-performance capacitive deionization of oxygenated saline water, *Mater. Horiz.* 2022, 9, 1708.

[S65] F. Meng, X. Tu, Y. Liu, K. Wang, X. Xu, X. Liu, Z. Gong, T. Lu and L. Pan, Carbon nanotube sustained ternary-metal Prussian blue analogues for superior-performance rocking-chair capacitive deionization, *Sep. Purif. Technol.* 2024, 329, 125155.

[S66] S. Choi, B. Chang, S. Kim, J. Lee, J. Yoon and J. W. Choi, Battery electrode materials with omnivalent cation storage for fast and charge-efficient ion removal of asymmetric capacitive deionization, *Adv. Funct. Mater.* 2018, 28, 1802665.

[S67] W. Xu, C. Tan, A. Wang, S. Hu, L. Deng, S. Boles, K. Sun, B. Li and H. Hu, Interlayer structure and chemistry engineering of MXene-based anode for effective capture of chloride anions in asymmetric capacitive deionization, *ACS Appl. Mater. Interfaces* 2023, 15, 16266.

[S68] Z. Guo, G. Shen, Z. Wang, Q. Ma, L. Zhang, B. Xiao, Y. Yan, Y. Zheng, Y. Liu and X. Yuan, Integrating FeOOH with bacterial cellulose-derived 3D carbon nanofiber aerogels for fast and stable capacitive deionization based on accelerating chloride insertion, *Desalination* 2024, 576, 117329.

[S69] L. Zhang, H. L. H. Chong, D. Luo, S. M. El-Bahy, P. Y. Moh, X. Xu and Z. M. El-Bahy, Facial synthesis of carbon nanotube interweaved FeOOH as chloride-insertion electrode for highly efficient faradic capacitive deionization, *Prog. Nat. Sci.-Mater. Int.* 2024, 34, 731.
